# Analysis of Tandem Repeats in Short‐Read Sequencing Data: From Genotyping Known Pathogenic Repeats to Discovering Novel Expansions

**DOI:** 10.1002/cpz1.70010

**Published:** 2024-11-05

**Authors:** Andreas Halman, Andrew Lonsdale, Alicia Oshlack

**Affiliations:** ^1^ Peter MacCallum Cancer Centre Melbourne Victoria Australia; ^2^ Sir Peter MacCallum Department of Oncology The University of Melbourne Victoria Australia; ^3^ School of Mathematics and Statistics The University of Melbourne Victoria Australia

**Keywords:** computational tools, genotyping, pathogenic mutations, rare diseases, short‐read sequencing, tandem repeats

## Abstract

Short tandem repeats (STRs) and variable‐number tandem repeats (VNTRs) are repetitive genomic sequences seen widely throughout the genome. These repeat expansions are currently known to cause ∼60 diseases, with expansions in new loci linked to rare diseases continuing to be discovered. Genome sequencing is an important tool for detecting disease‐causing variants and several computational tools have been developed to analyze tandem repeats from genomic data, enabling the genotyping and the identification of expanded alleles. However, guidelines for conducting the analysis of these repeats and, more importantly, for assessing the findings are lacking. Understanding the tools and their technical limitations is important for accurately interpreting the results. This article provides detailed, step‐by‐step instructions for three key use cases in STR analysis from short‐read genome sequencing data, which are also applicable to smaller VNTRs. First, it demonstrates an approach for genotyping known pathogenic loci and the identification of clinically significant expansions. Second, we offer guidance on defining tandem repeat loci and conducting genome‐wide genotyping studies, which is also applicable to diploid organisms other than humans. Third, instructions are provided on how to find novel expansions at loci not previously known to be associated with disease, aiding in the discovery of new pathogenic loci. Moreover, we introduce the use of newly‐developed helper tools that enable a complete and streamlined tandem repeat analysis protocol by addressing the gaps in current methods. All three protocols are compatible with human hg19, hg38, and the latest telomere‐to‐telomere (hs1) reference genomes. Additionally, this protocol provides an overview and discussion on how to interpret genotyping results. © 2024 The Author(s). Current Protocols published by Wiley Periodicals LLC.

**Basic Protocol 1**: Genotyping known pathogenic tandem repeat loci

**Alternate Protocol**: Genotyping known pathogenic tandem repeat loci with STRipy

**Support Protocol 1**: Installation of tools and ExpansionHunter catalog modification

**Basic Protocol 2**: Performing genome‐wide genotyping of tandem repeats

**Basic Protocol 3**: Discovering de novo tandem repeat expansions

**Support Protocol 2**: Compiling ExpansionHunter Denovo from source code and generating STR profiles

## INTRODUCTION

Short tandem repeats (STRs) are sequences composed of repetitions of short motifs, typically spanning up to 6 base pairs (bp) in length, while variable‐number tandem repeats (VNTRs) have longer motifs, with lengths of up to 100s of bps. Expansions of tandem repeat sequences at specific loci in the human genome have been linked to around 60 human diseases to date (STRs database, see Internet Resources). Analyzing such tandem repeats from short‐read sequencing data is challenging due to technical limitations and the inherent characteristics of these regions (Treangen & Salzberg, 2011). Several computational methods have been developed for genotyping STRs from genomic short‐read sequencing data (Dashnow et al., 2022; Dolzhenko et al., 2017; Mousavi et al., 2019), but there is a lack of guidelines for performing these STR analysis. This can lead to the exclusion of such analyses in patient data or misinterpretation of results. To address these knowledge gaps this article offers step‐by‐step instructions for addressing three pivotal use cases:

Genotyping known pathogenic loci (Basic Protocol 1). This protocol provides researchers with a method for genotyping known pathogenic STR loci. In addition to offering instructions on identifying potentially clinically significant repeat expansions, guidelines are provided for interpreting the results, with examples of true and false positive repeat expansions.
Performing genome‐wide genotyping of tandem repeats (Basic Protocol 2). This protocol enables researchers to define and genotype STR loci throughout the genome. By expanding STR analysis to encompass the entire genome, researchers can comprehensively catalogue STR variations. This protocol can be applied to humans as well as any other diploid organism with an available reference genome (including other animals and plants).
Discovering de novo STR expansions (Basic Protocol 3). This protocol describes steps to detect novel STR expansions in a sample or cohort at genomic locations that have not been previously associated with disease. Novel STR expansions may include repeat numbers significantly longer than the population alleles, including the possibility of inserting novel repeat sequences at a known STR locus. This is crucial for identifying new loci where an STR expansion might be linked to a disease.

**Figure 1:**
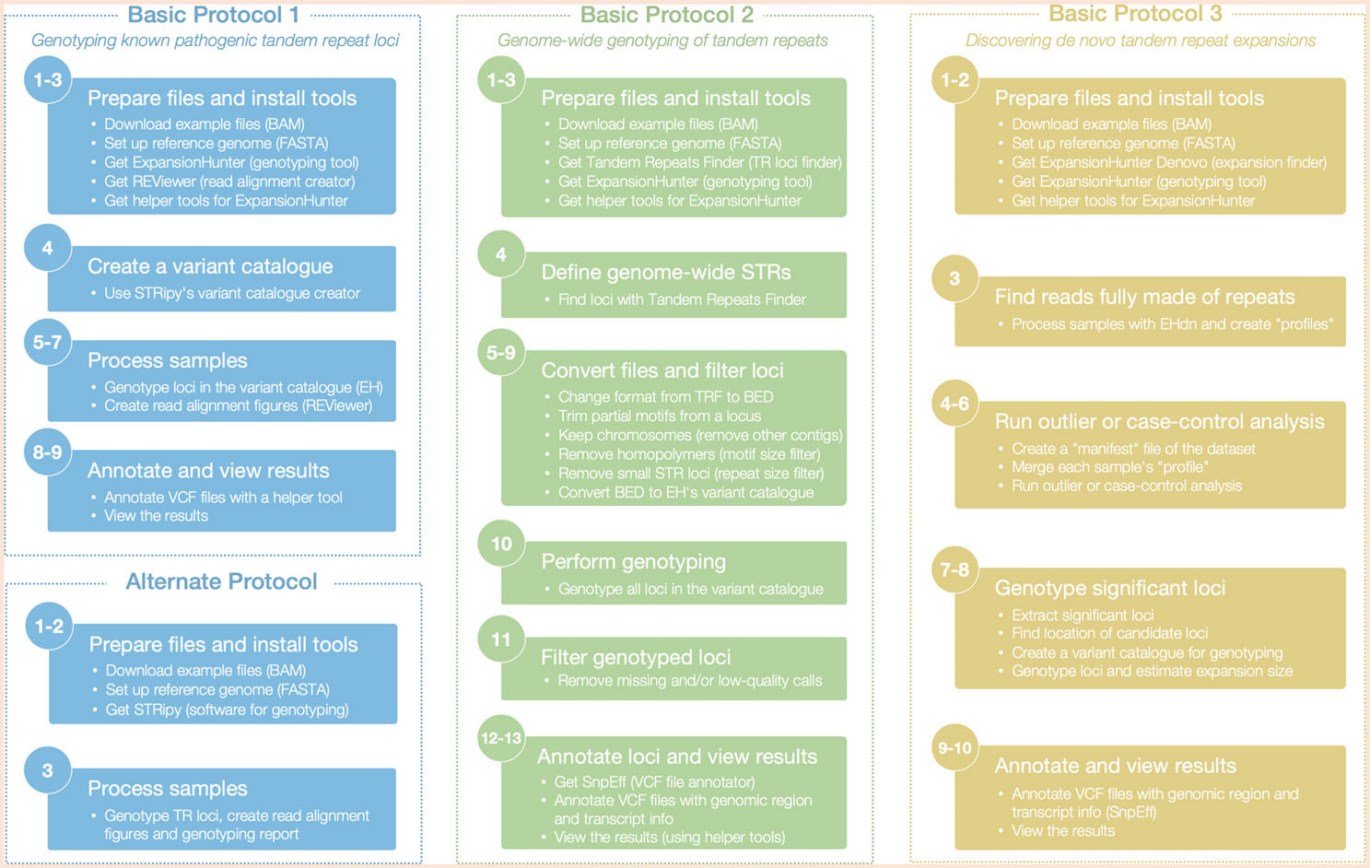
The workflow of the protocols. Summary of the steps for the Basic Protocols and Alternate Protocol are illustrated (within dashed rectangles), with a summary of the analysis steps presented inside filled rectangles. Step numbers are indicated within circles for each rectangle. TR, Tandem Repeat; TRF, Tandem Repeats Finder; EH, ExpansionHunter; EHdn, ExpansionHunter Denovo; VCF, Variant Call Format; BAM, Binary Alignment Map.

The significance of these protocols lies in their ability to provide clear, reproducible, and standardized methodologies for STR analysis using the common human reference genomes, such as hg19 and hg38, as well as the recent telomere‐to‐telomere genome reference (T2T CHM13v2.0/hs1). Moreover, Basic Protocols 2 and 3 can be applied to other diploid organisms that have a reference genome available. Although the examples focus on STRs, these protocols can also be applied to short VNTRs. See Figure 1 for an overview of the workflow.

## Basic Protocol 1 GENOTYPING KNOWN PATHOGENIC TANDEM REPEAT LOCI

This protocol details the steps for determining the genotype of known (pathogenic) STR loci in the human genome from Illumina's short‐read sequencing data (preferably PCR‐free). We also illustrate how to create read alignment visualizations to assess the quality of genotyping results. The tools utilized in this protocol, namely ExpansionHunter (Dolzhenko et al., 2017) and REViewer (Dolzhenko et al., 2022), have been specifically developed for this type of analysis. The protocol further provides examples of output results, along with their respective interpretations. Refer to Alternate Protocol for a simpler approach that achieves the same goal using our software called STRipy (Halman et al., 2022). This tool integrates and extends the functionalities of ExpansionHunter and REViewer. Provided as a Docker container, it does not require the installation of additional software.

### Necessary Resources

#### Hardware

Linux or MacOS 64‐bit operating system with adequate RAM, disk storage, and a network connection

#### Software

ExpansionHunter, https://gitlab.com/andreassh/ExpansionHunter (see note in the Background Information section in the Commentary)
REViewer, https://gitlab.com/andreassh/REViewer (see note in the Background Information section in the Commentary)
Samtools, https://github.com/samtools
Linux/macOS command line tools:
  wget
  unzip
*Instructions to install ExpansionHunter and REViewer are provided in this protocol and instructions to install Samtools and missing command line tools are provided in Support Protocol* *1*.

#### Files

Input files:
  Illumina short‐read sequencing files aligned to the hg19, hg38, or hs1 reference genome (in BAM or CRAM format), which must be sorted and indexed
  Corresponding reference genome used for alignment (in FASTA format)
  ExpansionHunter variant catalogue file (in JSON format)
Sample file:
  An example BAM file used in this protocol is available on Zenodo at http://doi.org/10.5281/zenodo.10872070

*NOTE*: In this protocol, we use the GRCh38 (hg38) reference genome which the provided example BAM file is aligned to. However, other versions of the hg38 reference genome are also compatible as long as the chromosome names begin with the “chr” prefix.

Create and go to a folder in your system where the steps of this protocol will be carried out. This folder will be referred to as the “tutorial” folder in this protocol. Inside it, tools will be stored in the “software” folder, the reference genome will be placed in the “reference” folder under the name “hg38.fa” and the example input BAM file is assumed to be located in the “examples/prot1” folder.

1 Prepare folders and download the example dataset.
  a. In the “tutorial” folder, create three folders to store the reference genome, tools, and example files:

mkdir reference software examples

  b. Set up a reference genome either by creating a symbolic link to it in the “reference” folder under the name “hg38.fa” (if it is available in the system), or by downloading it into the “reference” folder together with its index file:

wget -O reference/hg38.fa https://storage.googleapis.com/genomics-public-data/resources/broad/hg38/v0/Homo_sapiens_assembly38.fasta
wget -O reference/hg38.fa.fai https://storage.googleapis.com/genomics-public-data/resources/broad/hg38/v0/Homo_sapiens_assembly38.fasta.fai

    When copy/pasting the command, make sure there are no spaces in the URL.
  c. Download and unpack the example dataset:

 wget https://zenodo.org/records/10872070/files/examples.zip 
 unzip examples.zip && rm examples.zip

  d. Add the “software” folder into $PATH:
export PATH=$(pwd)/software:$PATH

  By adding the “software” folder containing the executable file to the $PATH, users can run the software by simply typing its name (e.g., ExpansionHunter) in the command line, rather than specifying the full or relative path. However, this change to $PATH is temporary and only remains in effect for the duration of the current bash session. To permanently add a folder to $PATH, refer to Support Protocol 1.
2 Install the tandem repeat genotyping tool ExpansionHunter (v5.0.0).
  Option A offers instructions for downloading the pre‐compiled binary for Linux, while Option B details the process of cloning the ExpansionHunter's repository and compiling the program (for both Linux and macOS). If Option A is unsuccessful, use Option B.
  a. Option A. Download pre‐compiled binary file.
    Download and unpack:


cd software
wget https://gitlab.com/andreassh/ExpansionHunter/-/releases/permalink/latest/downloads/binaries/ExpansionHunter
chmod +x ExpansionHunter

  The last command adds executable permission to the program; however, it may not always be necessary.
  b. Option B. Download and compile ExpansionHunter from source code.
  i. Download (clone) the repository:

cd software
git clone https://gitlab.com/andreassh/ExpansionHunter.git

  ii. Create a folder to save the executable program and compile it:

cd ExpansionHunter && mkdir build && cd build 
cmake ..
make

  Compiling a program requires the presence of several libraries in the system, refer to https://gitlab.com/andreassh/ExpansionHunter/‐/blob/master/docs/02_Installation.md for list of prerequisites.
  iii. Go back to the “software” folder, rename the ExpansionHunter installation folder (to separate it from the executable file) and move compiled binary to “software” folder with providing executable rights:

cd ../../
mv ExpansionHunter ExpansionHunter_installation
mv ExpansionHunter_installation/build/install/bin/ExpansionHunter ./
chmod +x ExpansionHunter

3 Install REViewer (v0.2.7) tool that will be used to create images of read alignments.
  Option A offers instructions for downloading the pre‐compiled binary, while Option B details the process of cloning the REViewer's repository and compiling the program. If Option A is unsuccessful, use Option B.
  a. Option A. Download pre‐compiled binary file into the “software” folder (Linux only).

wget https://gitlab.com/andreassh/REViewer/-/releases/permalink/latest/downloads/binaries/REViewer
chmod +x REViewer

  b. Option B. Download and compile REViewer (instructions apply both for Linux and macOS).
  i. Download (clone) the repository in the "software" folder:

git clone https://gitlab.com/andreassh/REViewer.git

  ii. Create a folder to store the build files and then compile the binary:

cd REViewer && mkdir build && cd build 
cmake ..
make

  iii. Go back to the “software” folder, rename the REViewer installation folder (to differentiate it from the executable file) and move compiled binary into the “software” folder:

cd ../../
mv REViewer REViewer_installation
mv REViewer_installation/build/install/bin/REViewer ./
chmod +x REViewer

4 Create a “variant catalogue” file.
  This is the file that contains all tandem repeat loci that the ExpansionHunter is going to genotype. The file is in JSON format, which could be created manually when the genomic coordinates and STR motif are known (see Support Protocol 1 for details on how to manually create or edit one). However, an easy and straightforward method to create a catalogue comprising known pathogenic loci is to use STRipy's (https://stripy.org) online resources that utilize a curated dataset of known pathogenic loci. The “ExpansionHunter's Catalog Creator” online tool (https://stripy.org/expansionhunter‐catalog‐creator) allows for the creation of a custom variant catalogue via a web interface. Nonetheless, the same task can be accomplished via the command line, as illustrated in this example.
  Go back to the “tutorial” folder and create the catalogue that contains information for genotyping HTT, DMPK, ATXN2 and ATXN10 loci that will be genotyped in the provided example files. Those loci are known to cause Huntington's disease, myotonic dystrophy 1, spinocerebellar ataxia 2 and 10, respectively.

cd ..
wget -O prot1_variant_catalogue.json https://api.stripy.org/catalogue/hg38/on/off/HTT,DMPK,ATXN2,ATXN10

  This action downloads a variant catalogue for the hg38 reference genome, which includes the HTT, DMPK, ATXN2 and ATXN10 loci, and saves it under the file name “prot1_variant_catalogue.json”. The last parameter in the URL is the list of loci that the catalogue contains. See https://stripy.org/database to find all loci IDs that can be used in the URL to include into the variant catalogue. Additionally, in the URL, after “catalogue/”, the reference genome is specified, followed by the choice of whether to use “chr” for chromosome notation (on) or not (off), and then by the decision to include (on) or exclude (off) off‐target regions (for genotyping long repeats exceeding read length). To generate a catalogue for the hg19 reference genome, replace “hg38” with “hg19” and to create a catalogue for the telomere‐to‐telomere reference genome, use “hs1”. In some cases, “‐‐no‐check‐certificate” might be required to add to the wget command. In this example, off‐target regions are not used. However, in the case of a long expansion (over the fragment length) and to obtain a more accurate estimation of the repeat size, they can be used, albeit with caution (see the Understanding Results section for more information).
5 Run ExpansionHunter on the aligned file (“examples/prot1/prot1_example.bam”) to genotype all loci in the variant catalogue (i.e., HTT, DMPK, ATXN2 and ATXN10).

ExpansionHunter --reads examples/prot1/prot1_example.bam --reference reference/hg38.fa --variant-catalog prot1_variant_catalogue.json --output-prefix prot1_example

  ExpansionHunter genotypes all loci specified in the variant catalogue in the provided BAM or CRAM file. Besides the catalogue and input BAM/CRAM file, it also requires the reference genome be compatible with the BAM/CRAM file. ExpansionHunter outputs a realigned BAM, VCF and JSON file, whose names are based on the “output‐prefix” parameter (e.g., “prot1_example_realigned.bam”, “prot1_example.vcf” and “prot1_example.json” as in this protocol).
6 Sort and index the ExpansionHunter's outputted BAM files with Samtools, which is required for the next step. See Support Protocol 1 for installation instructions for Samtools if that is not already in the system.

samtools sort prot1_example_realigned.bam > prot1_example_realigned.sorted.bam
samtools index prot1_example_realigned.sorted.bam

7 Run REViewer on the ExpansionHunter's output files to create visualizations of read alignments for each of the locus.

REViewer --reads prot1_example_realigned.sorted.bam --vcf prot1_example.vcf --reference reference/hg38.fa --catalog prot1_variant_catalogue.json --locus HTT,DMPK,ATXN2,ATXN10 --output-prefix prot1_example

  REViewer utilizes the realigned (and sorted) BAM and VCF files from ExpansionHunter to produce an image that shows read alignments for a specific tandem repeat locus. In some cases, such as when the repeated region exceeds the read length or when the confidence interval is large, it is important to view the read visualizations to assess the quality of the determined genotype. See the Understand section for instructions and tips.
8 Annotate the ExpansionHunter output VCF files with disease names and determine whether the repeat sizes in any of the alleles fall within the normal, intermediate, or pathogenic range.
  There are two options provided: Option A, using a Python script that performs the annotation on a local system; and Option B, utilizing an online VCF annotation tool, which does the annotation on‐the‐fly but requires uploading the VCF file. This may not be an option for protected clinical data.
  a. Option A. Use a local script. This requires Python3 to be accessible on your system.
  i. Clone a GitLab project named “Tools for ExpansionHunter” to the “software” folder. This project contains a script (“annotate_vcf.py”) that annotates VCF files produced by ExpansionHunter. This can be done in the “tutorial” folder:

git clone https://gitlab.com/andreassh/tools-for-expansionhunter.git software/tools-for-expansionhunter

  ii. Run the script to add annotations to the VCF file:

python3 software/tools-for-expansionhunter/annotate_vcf.py --input prot1_example.vcf --output prot1_example_ann.vcf

  Only loci whose name matches with the one in the STRipy's database (https://stripy.org/database) will get annotated. When a catalogue is created by using STRipy, the names will match.
  b. Option B. Use STRipy's VCF annotation online tool. Specify the input VCF file after the “file=@…” part and output file after the “>”:

curl -F 'file=@prot1_example.vcf' https://api.stripy.org/annotateVCF > prot1_example_ann.vcf

**Figure 2:**
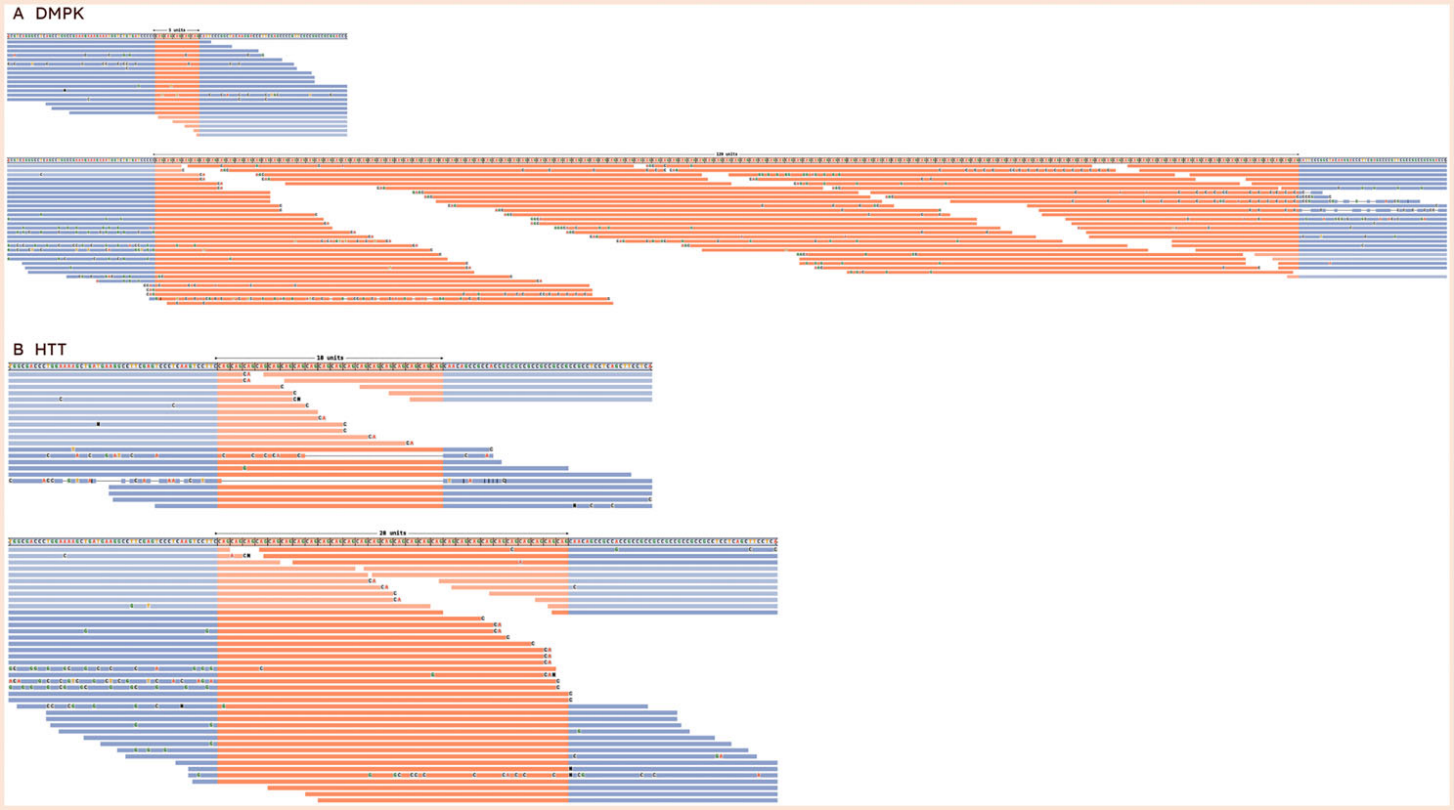
Read visualizations for DMPK and HTT loci. (**A**) Read alignments for the DMPK locus show support for a 5/129 genotype, and (**B**) alignments for the HTT locus show support for an 18/28 genotype.

**Figure 3:**
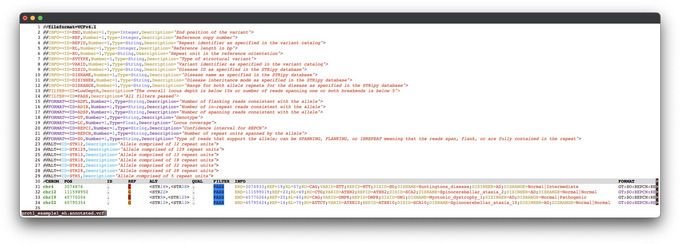
Annotated VCF file. Fields “DISID” (disease ID), “DISNAME” (disease name), “DISINHER” (disease inheritance) and “DISRANGE” (range within which the allele size falls, for both alleles) have been added during the annotation process.

**Figure 4:**
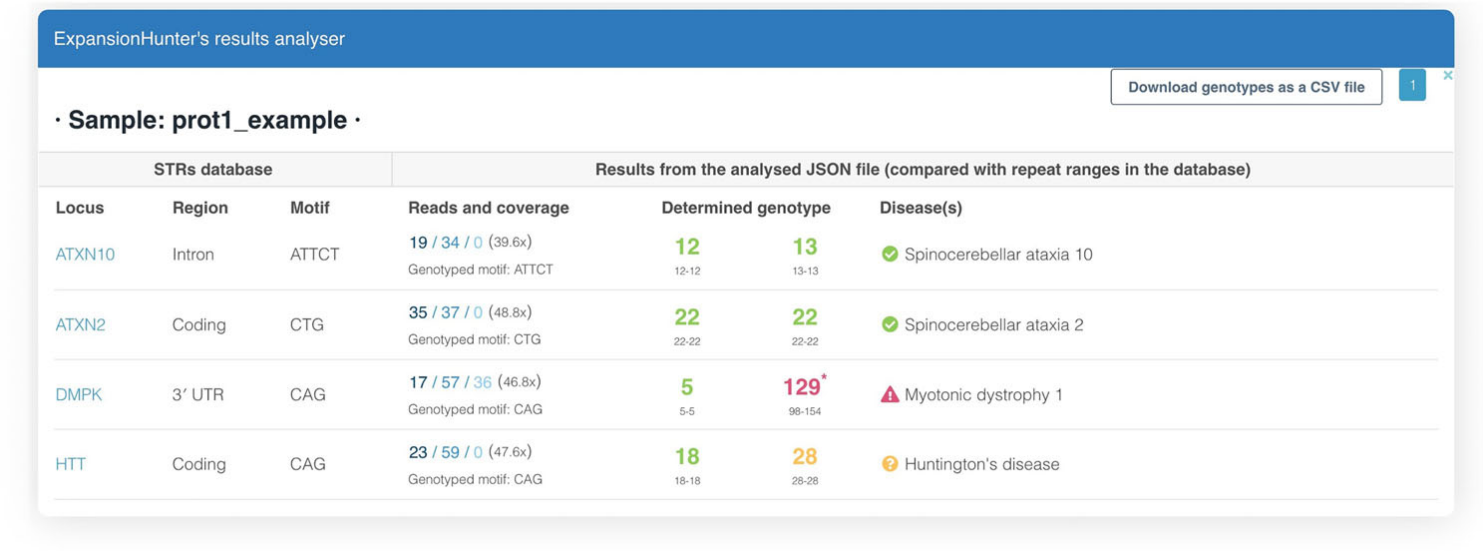
Report by STRipy's “ExpansionHunter results analyser” online tool. The number of repeats, as shown in the "Determined genotype" column, is color‐coded: green for the normal range, orange for the intermediate range, red for the pathogenic range, and grey for neither of the previous ranges, indicating “unknown”. Alleles that are population outliers (compared to the 1000 Genomes dataset, with *z* score ≥3.72) are marked with an asterisk.

  This option uses STRipy's online tool, which annotates the VCF on‐the‐fly and returns the annotated VCF. Only loci whose name matches with the one in STRipy's database will get annotated.
9 View the results.
  The successful completion of the above steps outputs the following files: a JSON file created by ExpansionHunter containing the results, a VCF file also containing the results and annotated with disease information, and SVG files of read alignment images for each locus created by REViewer. View the expected results (read alignment SVG images and annotated VCF file); "prot1_example.DMPK.svg", "prot1_example.HTT.svg" and "prot1_example_ann.vcf" are shown in Figures 2 and 3).
  Additionally, JSON files created by ExpansionHunter (e.g., the “prot1_example.json”) can be easily viewed using STRipy's “ExpansionHunter Results Analyser” online tool (https://stripy.org/expansionhunter‐results‐analyzer). This tool identifies the diseases associated with each locus and highlights the alleles, indicating whether they are in the normal, intermediate or pathogenic range and whether any of them are population outliers, resulting in a clear and printable report (as shown in Fig. 4).

## GENOTYPING KNOWN PATHOGENIC TANDEM REPEAT LOCI WITH STRipy

This alternative protocol facilitates the genotyping of any known pathogenic loci using the software STRipy, which utilizes ExpansionHunter and REViewer for genotyping and generating read visualizations as in Basic Protocol 1. STRipy includes several additions: besides providing a complete catalogue of STR loci for genotyping and creating an analysis report, it also has the capability to automatically determine off‐target regions where reads from long expansions can misalign. This provides an opportunity to genotype expansions longer than the read length for any locus. Additionally, it uses population data from the 1000 Genomes Project (1000 Genomes Project Consortium et al., 2015) to highlight population outliers, which can be important in determining potentially clinically significant loci when the determined allele size falls into the “unknown” range, instead of being classified as normal or pathogenic. As STRipy is also available as a Docker container, it can be used by those who lack the permissions to install software directly onto the systems they use. Employing STRipy achieves the same objectives as outlined in Basic Protocol 1.

### Necessary Resources

#### Hardware

Any operating system that supports Docker with adequate RAM, disk storage, and a network connection

#### Software

Docker, https://www.docker.com
STRipy (the “pipeline” version), https://gitlab.com/andreassh/stripy‐pipeline

#### Files

Input files:
  Illumina short‐read sequencing files aligned to the hg19, hg38, or hs1 reference genome (in BAM or CRAM format), which must be sorted and indexed
  The corresponding reference genome used for alignment (in FASTA format)

Sample file:
  An example BAM file used in this protocol is available on Zenodo at http://doi.org/10.5281/zenodo.10872070

*NOTE*: In this protocol, we use the GRCh38 (hg38) reference genome which the provided example BAM file is aligned on. However, other versions of the hg38 reference genome are also compatible as long as the chromosome names begin with “chr”. Refer to Support Protocol 1 for guidelines on downloading and preparing a reference genome if you do not have one.

This protocol shares the first step with Basic Protocol 1 (preparing folders and setting up the reference genome). If you have already completed this, proceed to step 2.

Create and go to a folder in your system where the steps of this protocol will be carried out. This folder will be referred to as the “tutorial” folder in this protocol. Inside it, tools will be stored in the “software” folder, the reference genome will be placed in the “reference” folder under the name “hg38.fa” and the example input BAM file is assumed to be located in the “examples/prot1” folder.

1 Prepare folders and download the example dataset.
  a. In the “tutorial” folder, create the folders to store the reference, tools, and example files:


mkdir reference software examples

  b. Set up a reference genome either by creating a symbolic link to it in the “reference” folder under the name “hg38.fa” (if it is available in the system), or by downloading it into the "reference" folder together with its index file: 


wget -O reference/hg38.fa https://storage.googleapis.com/genomics-public-data/resources/broad/hg38/v0/Homo_sapiens_assembly38.fasta
wget -O reference/hg38.fa.fai https://storage.googleapis.com/genomics-public-data/resources/broad/hg38/v0/Homo_sapiens_assembly38.fasta.fai

  c. Download and unpack the example dataset:

 wget https://zenodo.org/records/10872070/files/examples.zip 
 unzip examples.zip && rm examples.zip

2 Download/clone the latest version of STRipy into the “software” folder, make the script to run Docker as executable and create a folder to store output files.


git clone https://gitlab.com/andreassh/stripy-pipeline.git software/stripy
chmod +x software/stripy/runDocker.sh
mkdir stripy_results

  Ensure the “stripy_results” folder is added as a shared path in Docker to enable file writing.
3 Process samples with STRipy. 


./software/stripy/runDocker.sh -g hg38 -r reference/hg38.fa -l HTT,DMPK,ATXN2,ATXN10 -o stripy_results/ -i examples/prot1/prot1_example.bam

**Figure 5:**
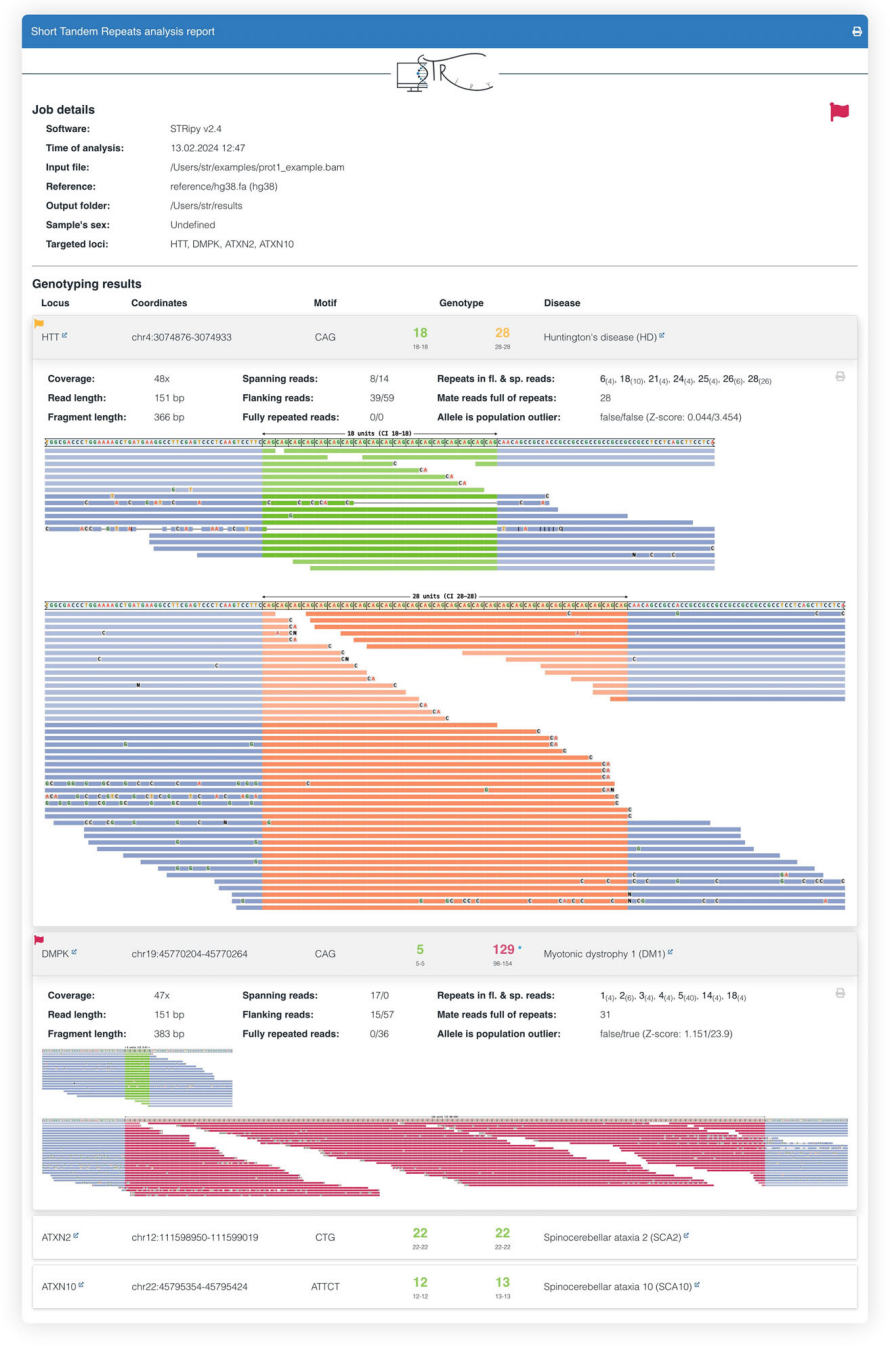
The output report of STRipy. Loci in the intermediate and pathogenic ranges are flagged, and alleles are color‐coded: green for the normal range, orange for the intermediate range, red for the pathogenic range, and grey for neither of the previous ranges, indicating “unknown”. Alleles that are population outliers (compared to the 1000 Genomes dataset, with *z* score ≥3.72) are marked with a dot on the top right of the repeat size.

  When the script is executed for the first time, a Docker image will be automatically built. This process likely takes several minutes, depending on your hardware and network speed. Once the image is constructed, it will be automatically used for subsequent runs. Refer to documentation at https://gitlab.com/andreassh/stripy‐pipeline for detailed description of parameters and configuration that can be used. In short: ‐g (genome, either hg19, hg38 or hs1), ‐r (reference genome file), ‐a (analysis mode; optional), ‐l (list of loci to genotype; optional), ‐c (custom BED file; optional) ‐o (output folder) and ‐i (input file). By default, the “standard” analysis mode is used, limiting genotyping to the fragment length (typically up to ∼300 bp). However, employing the parameter “‐a extended” switches the analysis mode, enabling the genotyping of loci with expansions beyond the fragment length (read the Understanding section to comprehend the limitations and risks associated with using this option).
4 View the results.
  By default, STRipy generates an HTML report file ("stripy_results/prot1_example.bam.html" report is shown in Fig. 5) containing results for all genotyped loci along with read alignment images for each locus whose repeat size falls outside the normal range (a default parameter setting which can be adjusted, refer to the tool's documentation).

## Support Protocol 1 INSTALLATION OF TOOLS AND ExpansionHunter CATALOG MODIFICATION

This support protocol provides instructions for installing the Samtools software, which is required for sorting and indexing BAM files, as well as for installing the “wget” Linux package for downloading files. It also includes instructions on how to permanently add a folder to the $PATH, enabling the use of tools on UNIX systems by typing their names instead of specifying the full folder path. Additionally, it explains the format of the ExpansionHunter variant catalogue, offering an explanation of its structure and instructions on how to modify it manually, if needed.

### Installation of Samtools

1 Download Samtools (v1.20) from its GitHub repository and unpack:

cd software/
wget https://github.com/samtools/samtools/releases/download/1.20/samtools-1.20.tar.bz2
tar -xf samtools-1.20.tar.bz2

2 Compile and install Samtools: 


cd samtools-1.20
./configure
make
sudo make install

  Note that the installation of Samtools requires administrator privileges (ability to use “sudo”) and should be available from command line. This can be verified by executing the “samtools” command. If this is not possible, follow the next two steps.
3 Move samtools binary file to the “software” folder (as for Basic Protocol 1). This assumes there is a folder named “software” in the same folder with “samtools‐1.20”.

cd ../../
mv software/samtools-1.20/samtools software/

4 Add the “software” folder into $PATH if it has not been done yet. 


export PATH=$(pwd)/software:$PATH

  To permanently add a folder to $PATH, see “Permanently add a folder into $PATH” below.

### Permanently add a folder into $PATH

The “export” command used on the command line is temporary and active for the active session only. To make it permanent, it must be included in the ~/.profile (sometimes also ~/.bash_profile) file for Linux or ~/.bash_profile file for macOS. Instead of using $(pwd), which represents the current system folder, the full path of the folder should be specified.

1 For example, to add “/home/user123/software” folder to $PATH, add the following line to the “.bash_profile” or “.profile” file: 


export PATH=/home/user123/software:$PATH

2 Save the file and run the following command to re‐run the profile file with the newly added command: 


source ~/.profile

  Use ~/.bash_profile instead of ~/.profile if the former was edited.
3 To add the current folder into $PATH and re‐run the profile file, simply run: 


 echo "export PATH=$PATH:$(pwd)" >> ~/.profile && source ~/.profile

  Change ~/.profile to ~/.bash_profile if you want to change the latter.

### Installation of packages not available on Linux

If “wget” is not available on your Linux system, it can typically be installed using a package manager tool, such as “apt” or “apt-get” in Debian‐based distributions (e.g., Debian and Ubuntu) or “yum” Red‐Hat based distributions (e.g., Red Hat Enterprise Linux, Fedora, CentOS). Instructions provided here are for using “apt-get”. Determine your Linux distribution and the available package manager to install it on your system if the following is not successful.

### Linux systems

If “wget” or “jq” is not available on your Linux system, it can typically be installed using a package manager tool, such as “apt” or “apt-get” in Debian‐based distributions (e.g., Debian and Ubuntu) or “yum” Red‐Hat based distributions (e.g., Red Hat Enterprise Linux, Fedora, CentOS). Instructions provided here are for using “apt-get”. Determine your Linux distribution and the available package manager to install it on your system if the following is not successful.

1 Update package indexes on system:


sudo apt-get update

2 If “wget” tool is not in the system, install it as follows:


sudo apt-get install wget

3 If “jq” tool is not in the system, install it as follows:


sudo apt-get install jq

### macOS systems

If “wget” or “jq” is not available on your macOS system, it can be easily installed using Homebrew, which is a package manager tool for macOS.

1 Follow the instructions on how to install Homebrew if it is not yet installed, from https://brew.sh.
2 Next, to Install “wget” and/or “jq”, run:


brew install wget
brew install jq

### ExpansionHunter variant catalogue modification

The variant catalogue of ExpansionHunter is in JSON format, where each locus is represented by a record within a list. For instance, the example below shows a catalogue entry that enables the tool to genotype the HTT and DMPK loci.


[
{
"LocusId": "DMPK",
"LocusStructure": "(CAG)*",
"ReferenceRegion": "chr19:45770204-45770264",
"VariantType": "Repeat"
},
{
"LocusId": "HTT",
"LocusStructure": "(CAG)*",
"ReferenceRegion": "chr4:3074876-3074933",
"VariantType": "Repeat"
}
]




The “LocusId” refers to the name of a locus, which in this example is the name of the gene containing the STR locus but can be another identifier as well. The “LocusStructure” represents the motif whose repeat number we aim to determine. This is often defined in enclosed parentheses followed by an asterisk (the asterisk indicates that the locus may contain zero or more repetitions of the motif). The “ReferenceRegion” specifies the coordinates of the repeated region within the reference genome (0‐based coordinates and chromosome name must match with names in the reference genome). Finally, the “VariantType” is typically set to “Repeat”. To search and use off‐target reads which allow the genotyping of alleles longer than the read length, “RareRepeat” value is used for the “VariantType” together with a list of genomic regions in “OfftargetRegions” key.

It is also possible to target multiple STR loci within a single record that are in close proximity to each other. For instance, the locus structure of ATXN8OS in the hg38 reference genome contains 10 repeats of the CTA motif followed by 15 repeats of the CTG motif. To provide genotypes for both loci, the repeat motifs for each locus should be added to the “LocusStructure” and both the “ReferenceRegion” and “VariantType” should be modified to be lists, containing coordinates for each locus listed. Additionally, the “VariantId” field can be used to custom‐name each listed locus; otherwise, the name will be derived from the LocusId and coordinates. 


[
{
"LocusId": "ATXN8OS",
"LocusStructure": "(CTA)*(CTG)*",
"ReferenceRegion": [
"chr13:70139353-70139383",
"chr13:70139383-70139428"
],
"VariantType": [
"Repeat",
"Repeat"
],
"VariantId": [
"ATXN8OS_CTA",
"ATXN8OS_CTG"
]
}
]




For guidance on utilizing off‐target regions and defining more advanced locus structures to genotype multiple repeats and/or include interruptions, please refer to the documentation at the project repository https://gitlab.com/andreassh/ExpansionHunter/‐/blob/master/docs/04_VariantCatalogFiles.md.

## Basic Protocol 2 PERFORMING GENOME‐WIDE GENOTYPING OF TANDEM REPEATS

This protocol details the process for identifying STR loci in a reference genome with Tandem Repeats Finder (TRF) tool and genotyping those loci using ExpansionHunter. It allows for establishing genome‐wide repeat lengths in the reference genome and determining allele length of a sample (with repeat sizes up to the fragment length). Because the hg38 reference genome contains numerous unassembled regions (Li & Freudenberg, 2014), not all TRF defined STR loci can be genotyped by ExpansionHunter (those adjacent to missing regions will be skipped), which therefore will be skipped. The instructions provided here focus on STRs (2 to 6 bp motifs), but the motif lengths can be easily adjusted to include repeats composed of longer motifs, such as smaller VNTRs.

### Necessary Resources

#### Hardware

Linux or MacOS 64‐bit operating system with adequate RAM, disk storage, and a network connection

#### Software

Tandem Repeats Finder, https://github.com/Benson‐Genomics‐Lab/TRF
ExpansionHunter, https://gitlab.com/andreassh/ExpansionHunter
SnpEff, https://pcingola.github.io/SnpEff/download (optional)
Java, https://www.oracle.com/java/ (optional)
Linux/macOS command line tools:
  wget
  unzip
  jq

#### Files

Input files:
  Illumina short‐read sequencing files aligned to the hg19, hg38 or hs1 reference genome (in BAM or CRAM format), which must be indexed
  The corresponding reference genome used for alignment (in FASTA format)

Sample file:
  Samples files used in all protocols are available on Zenodo at http://doi.org/10.5281/zenodo.10872070

*NOTE*: In this protocol, we use the GRCh38 (hg38) reference genome, which the provided example BAM file is aligned to. Other versions of reference genomes may cause errors due to incompatibility of chromosome names between the example file and files created based on the reference genome. Refer to Support Protocol 1 for guidelines on downloading and preparing a reference genome if you do not have one.

This protocol shares the first step with Basic Protocol 1 (preparing folders). If you have already completed this, proceed to step 2.

Create and go to a folder in your system where the steps of this protocol will be carried out. This folder will be referred to as the “tutorial” folder in this protocol. Inside it, tools will be stored in the “software” folder, the reference genome will be placed in the “reference” folder under the name “hg38.fa” and the example input BAM file is assumed to be in the “examples/prot2” folder.

1 Prepare folders and download the example dataset.
  a. In the “tutorial” folder, create the folders to store the reference, tools, and example files: 


mkdir reference software examples

  b. Set up a reference genome either by creating a symbolic link to it in the “reference” folder under the name “hg38.fa” (if it is available in the system), or by downloading it into the "reference" folder together with its index file:


wget -O reference/hg38.fa https://storage.googleapis.com/genomics-public-data/resources/broad/hg38/v0/Homo_sapiens_assembly38.fasta
wget -O reference/hg38.fa.fai https://storage.googleapis.com/genomics-public-data/resources/broad/hg38/v0/Homo_sapiens_assembly38.fasta.fai

  c. Download and unpack the example dataset: 


 wget https://zenodo.org/records/10872070/files/examples.zip 
 unzip examples.zip && rm examples.zip

  d. Add the “software” folder into $PATH: 


export PATH=$(pwd)/software:$PATH

  By adding the “software” folder containing the executable file to the $PATH, users can run the software by simply typing its name (e.g., ExpansionHunter) in the command line, rather than specifying the full or relative path. However, this command is temporary and works until the current bash session is open. To permanently add a folder to $PATH, refer to Support Protocol 1.
2 Install STRs genotyping tool ExpansionHunter. Refer to Basic Protocol 1 step 2 for installation instructions.
3 While in the “tutorial” folder, download Tandem Repeats Finder (TRF) version 4.09.1 for Linux (Option A) or macOS (Option B) and make the file executable.
  a. Option A. Installation for Linux:


wget -O software/trf409.legacylinux64 https://github.com/Benson-Genomics-Lab/TRF/releases/download/v4.09.1/trf409.legacylinux64
chmod +x software/trf409.legacylinux64

  b. Option B. Installation for macOS:


wget -O software/trf409.macosx https://github.com/Benson-Genomics-Lab/TRF/releases/download/v4.09.1/trf409.macosx
chmod +x software/trf409.macosx

4 Run TRF to identify genome‐wide STR loci and save them into a file.


./software/trf409.legacylinux64 reference/hg38.fa 2 100 100 80 10 30 6 -h -d -ngs > str_loci.trf

  This command identifies all STRs up to 6 bp in length and regions composed (mostly) of consecutive repeats. Note that TRF raw output has coordinates for loci that often ends with an incomplete motif (e.g., CAGG‐CAGG‐CA); those incomplete loci will be trimmed in the next step. Refer to the Critical Parameters section for insights on TRF settings and how to determine repeats with motifs longer than 6 bp. For macOS, use “./software/trf409.macosx”. Note that running TRF may take time (several hours). Example materials also include “str_loci_normChr_2‐6bp_5rep.bed” file in the “examples/prot2” folder that allows skipping steps from 3 to 8, but requires to copy the file to the “tutorial” folder beforehand (run: cp examples/prot2/str_loci_normChr_2‐6bp_5rep.bed ./).
5 Convert the “str_loci.trf” file, which is in TRF format, to a BED format (using 0‐based coordinates) for easier processing, retaining the chromosome name, start and end coordinates of the region, motif, and number of repeats in the reference genome.
  This command also trims STR loci coordinates to end with a whole repeat (e.g., in a determined locus with CAGG‐CAGG‐CA sequence, the residual CA will be removed, and the coordinates will then correspond to CAGG‐CAGG region, containing full repeats).


 awk -v OFS='\t' '/^@/{name=substr($0, 2); sub(/ .*/, "", name); next} {motif_len=length ($14); int_repeat=int($4); $2=$1-1; $3=$2+int_repeat*motif_len; $4=name; $5=int_repeat; print $4, $2, $3, $14, $5;}' str_loci.trf > str_loci.bed

6 Keep all loci where the chromosome name contains only letters and numbers (e.g., chr5, X, chrY) and remove others (e.g., chr17_KI270729v1_random, HLA‐DRB1*16:02:01, chrEBV, etc., which can cause issues in genotyping when the sample file does not have those contigs).
  This step is optional and can be skipped if the reference genome does not have chromosomes named in this manner (e.g., uses NCBI reference sequence IDs) or it is necessary to genotype those regions (e.g., chrM).


 awk -v OFS='\t' '$1 ~ /^(chr)?[0-9XY]{1,2}$/ && $2 ~ /^[0-9]+$/ && $3 ~ /^[0-9]+$/'  str_loci.bed > str_loci_normChr.bed

  To include mitochondria, add “M” after the “0‐9XY” section to form “0‐9XYM”, allowing to keep contigs starting with “chrM” or “M” (in addition to 0‐9, X and Y). Note that genotyping such small contigs can lead to errors when the STR locus is located at one end or the other of the chromosome.
7 Remove homopolymers and retain regions with motif sizes ranging from 2 to 6 bp in length (optional). Use “str_loci.bed” as an input if step 6 was skipped.


awk -F'\t' 'length($4) >= 2 {print}' str_loci_normChr.bed > str_loci_normChr_2-6bp.bed

  We use equal or greater (>=) than 2 to define minimum length of motif sizes. Since TRF was run with a maximum motif size of 6 bp, we do not need to use a parameter to define the upper length here. For example, to retain only trinucleotides, use: “length($4) == 3”; for loci with motif lengths between 3 to 6, use “length($4) >= 3 && length($4) <= 6”.
8 Filter out small STR loci, such as those that contain <5 repeats (optional). If any of the last steps were skipped, choose the last created BED file as input.


awk -F'\t' '$5 >= 5 {print}' str_loci_normChr_2-6bp.bed > str_loci_normChr_2-6bp_5rep.bed

  The parameters chosen, and the length of loci included in the analysis, ultimately depend on the user's preference. In this example, we stipulate a minimum of 5 repeats in the reference genome locus. The number of repeats is in the fifth column that is denoted as $5 in the command. For example, to filter out all regions with fewer than 10 repeats, use $5 >= 10 (i.e., value in the fifth column is required to be ≥10).
9 Convert the final BED file (from the last step you completed, such as “str_loci_normChr_2‐6bp_5rep.bed”) to ExpansionHunter's variant catalogue file. To do this locally, we need to use a command‐line tool “jq”, which may need to be installed to the system (see Support Protocol 2).


awk -F'\t' '{printf "{\"LocusId\": \"%s-%s-%s\", \"LocusStructure\": \"(%s)*\", \"ReferenceRegion\": \"%s:%s-%s\", \"VariantType\": \"Repeat\"}\n", $1, $2, $3, $4, $1, $2, $3}' str_loci_normChr_2-6bp_5rep.bed | jq -s . > eh_catalogue_2-6bp_5rep.json

  This command utilizes the ‘jq’ tool, which is designed for parsing and manipulating JSON‐formatted data from the command line. It takes the location and repeat motif from the BED file, parses them into JSON format, and then saves them as the variant catalogue file. Note that to use the BED file provided with the example dataset, copy the “examples/prot2/str_loci_normChr_2‐6bp_5rep.bed” file to the “tutorial” folder.
10 Perform a genome‐wide genotyping on a sample (BAM or CRAM file) with ExpansionHunter:


ExpansionHunter --reads examples/prot2/prot2_example.bam --reference reference/hg38.fa --variant-catalog eh_catalogue_2-6bp_5rep.json --output-prefix prot2_example --threads 6 --analysis-mode streaming

  In this example of using ExpansionHunter, we employ the “‐‐threads” parameter to allocate more processor threads, improving the speed of the analysis (6 in this example, adjust if needed). Given our extensive genome‐wide repeat catalogue, we opt for the “‐‐analysis‐mode streaming” parameter. This mode reads in the entire BAM file (which may be unsorted) and conducts the analysis more rapidly than the default “seeking” mode (requires a sorted file), though it demands significantly more RAM (likely >64 GB, depending on the sample). Note that by default, the “sex” parameter is set to “female”, which results in STR loci on chromosome X being interpreted as diplotypes. When analyzing male samples, it is essential to specify the sex to ensure haploid genotypes are obtained for loci on chromosome X (See Critical Parameters regarding ExpansionHunter in the Commentary section). For the example aligned file, we use the same file as in Basic Protocol 1. Note that it is expected to encounter numerous warning messages for hg19 and hg38 reference genomes, which refer to the inability to genotype many loci defined by TRF (those where repeats are located close to a chromosome edge or in unsequenced parts of the reference genome).
11 Filter the VCF file by removing loci with missing results or those with low coverage (fewer than 10 repeats) that did not pass the filter set by ExpansionHunter.


 awk -v OFS='\t' '($7 == "PASS") || /^#/' prot2_example.vcf > prot2_example_pass.vcf

12 Optional. Annotate VCF files to provide information about the closest genomic regions to the locus.
  Running this protocol on an actual biological sample will likely yield numerous results, and we may want to prioritize certain loci, such as those in exons or UTRs, while filtering out others. To achieve this, we must annotate each locus in the VCF files. Among the tools available for this purpose, this protocol uses a popular tool called SnpEff. While this tool is primarily designed for annotating variants/INDELs, it also offers a feature that identifies the proximity to the nearest genomic regions and provides relevant details, including distance to the region, the region's type (e.g., exon, intron), gene name, and transcript ID. Note that SnpEff requires Java to be installed on the system.
  a. Download and unpack the latest version of SnpEff (https://pcingola.github.io/SnpEff). For advanced installation/configuration options and documentation, refer to the provided link. 


cd software/
wget https://snpeff.blob.core.windows.net/versions/snpEff_latest_core.zip
unzip snpEff_latest_core.zip

  b. Download a pre‐built database for the used genome (hg38 in this example), as required by SnpEff. Since database builds can change over time, we need to determine which databases are currently available before downloading one.
  i. View available databases for humans: 


cd snpEff
java -jar snpEff.jar databases | grep -i "Homo_sapiens"

    Instead of “Homo_sapiens” you can also use other species' full names or fragments of names, e.g., “Mus_musculus” for mouse, “Vitis_vinifera” or just “vinifera” for grape vine.
  ii. Download the database (we are using “GRCh38.99” since it is the latest version available at the time this protocol was written). 


java -jar snpEff.jar download -v GRCh38.99

  c. Go back to the “tutorial” folder and annotate the VCF file produced in the previous step. 


cd ../..
java -jar software/snpEff/snpEff.jar closest -ud 0 GRCh38.99 prot2_example_pass.vcf > prot2_example_pass_ann.vcf

**Figure 6:**
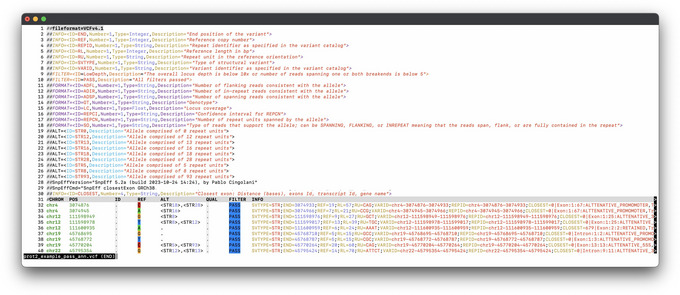
SnpEff annotated genotyping results (VCF). After the “CLOSEST” tag the distance to the closest region and transcripts in that region this STR locus is overlapping are shown.

  Use the same genome version that you have previously downloaded (e.g., “GRCh38.99”). We use “‐ud 0” here that disables upstream and downstream annotations, thereby reporting distance to only exons, introns, and UTRs. See Figure 6 for the results. The annotated VCF file contains distance (in base pairs) to the closest genomic region together with the transcript and gene IDs/names.

13 Optional. Show all annotations for each locus.
  Annotations in the VCF file are not easily readable by humans and require an additional tool or script for processing. Here, we show one option, which is using a simple script (available in the “Tools for ExpansionHunter” repository) to read in each locus and display all transcripts that the STR locus is part of.
  a. Get the “Tools for ExpansionHunter” repository and make the script that is being used here as executable: 


git clone https://gitlab.com/andreassh/tools-for-expansionhunter.git software/tools-for-expansionhunter
chmod +x software/tools‐for-expansionhunter/extractSnpeffAnn.sh

  b. Run the script with the VCF file name as the first parameter. The second (optional) parameter specifies the minimum repeat size in base pairs for any of the alleles, and the third (optional) parameter indicates whether the STR locus has to be within the specified region (exon or intron). For example, to show all results, use: 


./software/tools-for-expansionhunter/extractSnpeffAnn.sh prot2_example_pass_ann.vcf >prot2_example_pass_ann_all.tsv

  c. To show only those with repeat size of at least 100 bp that are located in exons, use: 


./software/tools-for-expansionhunter/extractSnpeffAnn.sh prot2_example_pass_ann.vcf 100Exon > prot2_example_pass_ann_100bp_exons.tsv

**Figure 7:**
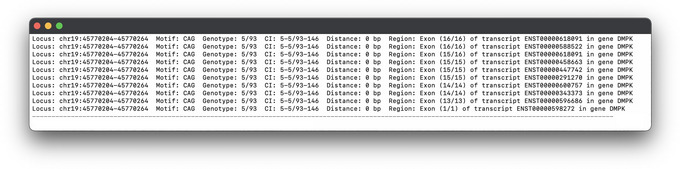
Results showing all transcripts for the only locus where length is at least 100 bp and is located in an exon.

  View the “prot2_example_pass_ann_100bp_exons.tsv” file or see Figure 7 for results.

14 Conduct further analysis.
  This protocol generates “prot2_example_pass_ann.vcf” and “prot2_example_pass_ann_100bp_exons.tsv” files as the final output and subsequent analysis can now be conducted based on the research question, such as identifying expanded alleles, and comparing samples or cohorts. The expansions identified here are limited by the fragment length. For detecting the presence of any longer (de novo) expansions in the genome, refer to Basic Protocol 3.

## Basic Protocol 3 DISCOVERING DE NOVO TANDEM REPEAT EXPANSIONS

This protocol describes a method for conducting a genome‐wide search for novel expanded repeats and performing a case/control or outlier analysis to identify cases that diverge from the normal population. We are utilizing ExpansionHunter Denovo (EHdn) to find expansions of STRs and lower end of VNTRs (2 to 20 bp) from a set of short‐read sequencing BAM/CRAM files (Dolzhenko et al., 2020). For optimal results, it is recommended by the authors of the tool that the set of samples are prepared with PCR‐free method and are sequenced using the same instrument with a similar coverage of at least 30×. Additionally, all data should be aligned using the same aligner, ideally without post‐processing steps, such as INDEL realignment or recalibration. The dataset should include one or more samples to test for repeat expansions, along with a set of control samples assumed to not have pathogenic expansions. Including a larger number of control samples in the analysis improves the statistical power to detect repeat size deviations from the normal population. Finally, we attempt to determine the size of the repeats by using ExpansionHunter on the significant loci and annotating the selected loci with genomic information.

### Necessary Resources

#### Hardware

Linux or MacOS 64‐bit operating system with adequate RAM, disk storage, and a network connection

#### Software

ExpansionHunter Denovo, https://gitlab.com/andreassh/ExpansionHunterDenovo (see note in the Background Information section in the Commentary)
ExpansionHunter, https://gitlab.com/andreassh/ExpansionHunter
Python 3, https://www.python.org
Linux/macOS command line tools:
  wget
  unzip
  jq

#### Files

Input files:
  Illumina short‐read sequencing files aligned to the hg19, hg38, or hs1 reference genome (in BAM or CRAM format), which must be sorted and indexed
  The corresponding reference genome (in FASTA format) used for alignment
Sample file:
  Sample files used in this protocol are available on Zenodo at http://doi.org/10.5281/zenodo.10872070

*NOTE*: In this protocol, we use the GRCh38 (hg38) reference genome, which the provided example BAM file is aligned on. Refer to Support Protocol 1 for guidelines on downloading and preparing a reference genome if you do not have one.

This protocol shares the first step with Basic Protocol 1 and 2 (preparing folders). If you have already completed this, proceed to step 2.

Create and go to a folder in your system where the steps of this protocol will be carried out. This folder will be referred to as the “tutorial” folder in this protocol. Inside it, tools will be stored in the “software” folder, the reference genome will be placed in the “reference” folder under the name “hg38.fa” and the example input BAM file is assumed to be in the “examples/prot3” folder.

1 Prepare folders and download the example dataset.
  a. In the “tutorial” folder, create the folders to store the reference, tools, and example files: 


mkdir reference software examples

  b. Set up a reference genome either by creating a symbolic link to it in the “reference” folder under the name “hg38.fa” (if it is available in the system), or by downloading it into the "reference" folder together with its index file: 


wget -O reference/hg38.fa https://storage.googleapis.com/genomics-public-data/resources/broad/hg38/v0/Homo_sapiens_assembly38.fasta
wget -O reference/hg38.fa.fai https://storage.googleapis.com/genomics-public-data/resources/broad/hg38/v0/Homo_sapiens_assembly38.fasta.fai

  c. Download and unpack the example dataset: 


 wget https://zenodo.org/records/10872070/files/examples.zip 
 unzip examples.zip && rm examples.zip

  d. Add the “software” folder into $PATH: 


export PATH=$(pwd)/software:$PATH

  By adding the “software” folder containing the executable file to the $PATH, users can run the software by simply typing its name (e.g., ExpansionHunter) in the command line, rather than specifying the full or relative path. However, this command is temporary and works until the current bash session is open. To permanently add a folder to $PATH, refer to Support Protocol 1.
2 Download the compiled binary of ExpansionHunter Denovo for Linux along with companion scripts for statistical analysis. Then, move the binary to the “software” folder and make it executable. For instructions on how to compile it from source code and/or install it on macOS, see Support Protocol 3. 


cd software
wget https://gitlab.com/andreassh/ExpansionHunterDenovo/-/releases/permalink/latest/downloads/bundles/ExpansionHunterDenovo_dir.zip
unzip ExpansionHunterDenovo_dir.zip
mv ExpansionHunterDenovo_dir/ExpansionHunterDenovo ./
chmod +x ExpansionHunterDenovo
rm ExpansionHunterDenovo_dir.zip

3 Go back to the “tutorial” folder and create “STR profiles” for each BAM file found in the “examples/prot3/” folder (into the “str‐profiles/” folder), which contains information about any long expansions of 2 to 20 bp motifs. Also, a “prot3_manifest.tsv” file will automatically be created, in which each sample/file is defined (required in step 5). 


cd ../
mkdir str-profiles
> prot3_manifest.tsv
shopt -s nullglob
for sample_path in examples/prot3/*.{bam,cram}; do
extension="${sample_path##*.}"
sample_name=$(basename "$sample_path" ".$extension")
ExpansionHunterDenovo profile \
--reads "$sample_path" \
--reference reference/hg38.fa \
--max-irr-mapq 60 \
--output-prefix str-profiles/"$sample_name"
echo -e "${sample_name}\tcase/control\tstr-profiles/${sample_name}.str_profile.json" >> prot3_manifest.tsv
done
shopt -u nullglob

  Make sure to copy all text from “for sample_path…” until the “done” line, as this is one command. This script generates an STR profile file for each sample (BAM file) found in the “examples/prot3/” folder (str_profile.json file). Here, we use the “‐‐max‐irr‐mapq” parameter to change the value from the default (40) to 60 in order to include in‐repeat reads with higher mapping quality (see the Critical Parameters section for an explanation). Also, refer to Support Protocol 3 to see how to generate profiles for only one sample, if required.
4 Decide the type of your analysis and prepare the manifest file.
  ExpansionHunter Denovo incorporates scripts to perform two types of analysis: (A) case‐control and (B) outlier. A case‐control analysis (A) is suitable when it is anticipated that a substantial portion of the cases will have expansions of the same repeat. For instance, when this analysis is applied to a dataset comprising spinocerebellar ataxia 27B patients and healthy individuals, the expectation is to identify the GAA repeat in the FGF14 gene as significant. If the cases are made of samples from patients having a variety of phenotypes, it may be reasonable to presume that there is not an enrichment of any particular expansion, rendering the case‐control analysis unsuitable. Under these circumstances, an outlier analysis (B) could be used to identify repeats that are expanded in a small subset of cases.
  The command ran in step 3 already created a new file “prot3_manifest.tsv”, which describes the dataset. The manifest file includes columns for the sample identifier, case‐control status, and the path to the corresponding STR profile for each sample (note that the file is tab‐delimited). The automatically created file has sample ID derived from filename, includes “case/control” text that needs to be edited and the file location. Edit the manifest file (e.g., nano prot3_manifest.tsv) and mark which samples are controls and which ones are cases. In our example, the final file content must be as follows:

**Table:** 

sampleA	control	str-profiles/sampleA.str_profile.json
sampleB	control	str-profiles/sampleB.str_profile.json
sampleC	control	str-profiles/sampleC.str_profile.json
sampleD	control	str-profiles/sampleD.str_profile.json
sampleE	control	str-profiles/sampleE.str_profile.json
sampleF	control	str-profiles/sampleF.str_profile.json
sampleG	control	str-profiles/sampleG.str_profile.json
sampleH	control	str-profiles/sampleH.str_profile.json
sampleI	control	str-profiles/sampleI.str_profile.json
sampleJ	control	str-profiles/sampleJ.str_profile.json
sampleK	control	str-profiles/sampleK.str_profile.json
sampleL	control	str-profiles/sampleL.str_profile.json
sampleM	control	str-profiles/sampleM.str_profile.json
sampleN	control	str-profiles/sampleN.str_profile.json
sampleO	control	str-profiles/sampleO.str_profile.json
sampleXL	case	str-profiles/sampleXL.str_profile.json
sampleXXL	case	str-profiles/sampleXXL.str_profile.json
5 Run ExpansionHunter Denovo to merge STR profiles.


ExpansionHunterDenovo merge --reference reference/hg38.fa --manifest prot3_manifest.tsv --output‐prefix prot3

  The created multisample STR profile is titled “prot3.multisample_profile.json”. This file encompasses data on long repeats (exceeding the read length) throughout the genome and includes specific details for each sample, such as read length and depth of read coverage.
6 Run case‐control (Option A) or outlier analysis (Option B).
  Both options involve computing statistics and comparing STR lengths between case and control samples defined in the manifest file. There are two supported methods for this comparison: a locus‐based comparison and a motif‐based comparison. These comparisons are executed using the Python 3 scripts (casecontrol.py or outlier.py), which are found in the scripts/ directory of ExpansionHunter Denovo.
  a. Option A. Case‐control analysis.
  i. Selection 1. Locus based comparison. The locus‐based comparison can differentiate repeats that are longer than the read length but shorter than the fragment length. It can be run as follows: 


./software/ExpansionHunterDenovo_dir/scripts/casecontrol.py locus --manifest prot3_manifest.tsv --multisample-profile prot3.multisample_profile.json --output prot3.casecontrol_locus.tsv

    The file titled “prot3.casecontrol_locus.tsv” includes several columns. The “contig” column indicates the contig of the repeat region, the “start” and “end” columns specify the approximate start and end of the repeat, and the “motif” column shows the inferred repeat motif. Finally, the “pvalue” column displays the p value from the Wilcoxon rank‐sum test, the “bonf_pvalue” lists the p value after Bonferroni correction and the “counts” column provides depth‐normalized counts of anchored in‐repeat reads for each sample, omitting those samples that have a zero count.
  ii. Selection 2. The motif‐based comparison focuses on assessing the enrichment of genomes with repeats that exceed the fragment length. It can be executed in the following manner: 


./software/ExpansionHunterDenovo_dir/scripts/casecontrol.py motif --manifest prot3_manifest.tsv --multisample‐profile prot3.multisample_profile.json --output prot3.casecontrol_motif.tsv

    The file named “prot3.casecontrol_motif.tsv” contains columns for each sample. The “motif” column indicates the identified repeat motif, the “pvalue” column presents the p value obtained from the Wilcoxon rank‐sum test, the “bonf_pvalue” column shows the p value after applying the Bonferroni correction, and lastly, the “counts” column contains the depth‐normalized counts of in‐repeat read pairs for each sample, excluding those samples that have a zero count.
  b. Option B. Outlier analysis.
  i. Selection 1. Locus based comparison. The locus‐based comparison can differentiate repeats that are longer than the read length but shorter than the fragment length. It can be run as follows: 

./software/ExpansionHunterDenovo_dir/scripts/outlier.py locus --manifest prot3_manifest.tsv --multisample-profile prot3.multisample_profile.json --output prot3.outlier_locus.tsv

    The “prot3.outlier_locus.tsv” file contains the following columns: the “contig”, “start” and “end” columns indicate the contig with approximate start and end of the repeat, respectively. The “motif” column shows the inferred repeat motif, the “top_case_zscore” column provides the highest z score for a case sample. In the “high_case_counts” column, the counts of case samples with a z score >1.0 are listed and finally, the “counts” column includes all nonzero counts for the samples.
  ii. Selection 2. The motif‐based comparison focuses on assessing the enrichment of genomes with repeats that exceed the fragment length. It can be executed in the following manner: 


./software/ExpansionHunterDenovo_dir/scripts/outlier.py motif --manifest prot3_manifest.tsv --multisample-profile prot3.multisample_profile.json --output prot3.outlier_motif.tsv

  The file named “prot3.outlier_motif.tsv” contains the following columns: the “motif” column shows the inferred repeat motif, the “top_case_zscore” column provides the highest z score observed in a case sample, the “high_case_counts” column enumerates the number of case samples that have a z score >1.0 and lastly, the “counts” column gives the nonzero counts for all the samples included in the dataset.
7 The output files from the analysis can be manually examined to identify any loci where a significantly higher number of in‐repeat reads (completely composed of the repeat motif) were found, indicating a long repeat size. In this optional step, we aim to determine the size of an expansion more accurately by using the genotyping tool ExpansionHunter on the significant loci.
  The created *locus.tsv files contain approximate locations in the genome of the STR region with the expansion but may be a few thousand base pairs away from it. The values in “contig”, “start”, “end”, and “motif” columns enable us to find repeated loci in the reference genome and then use a targeted tandem repeat genotyper to size the repeat. The fifth column of these files contains either the case *z* score (for outlier analysis) or the *p* value (for case‐control analysis), which will be used to identify only statistically significant loci. However, the lack of statistical significance does not necessarily imply the absence of an expansion, as the statistical significance is relative to the control dataset.
  a. Clone the GitLab project named “Tools for ExpansionHunter” (unless done already) and move it to the “software” folder (this was also utilized in Basic Protocol 1 and 2).
  This folder contains a script (findLocus.py) that can read in *locus.tsv files, use the approximate coordinates and the repeated motif provided for each locus to find repeated regions in the reference file. Subsequently, it automatically creates the variant catalogue file for ExpansionHunter, which is used for genotyping the locus. While this is a straightforward approach suitable for repeats found in the reference genome, it may not be effective for “nested” or “replaced types” of repeats, which contain a novel motif that is not present in the reference genome. For rigorous analysis, we recommend manually examining the *locus.tsv output files to ensure no details are overlooked.


git clone https://gitlab.com/andreassh/tools-for-expansionhunter.git software/tools-for-expansionhunter
  b. Running a script in this protocol requires Python 3 to be accessible on your system via "python3" command, as well as the libraries “regex” and “pysam”, which may not be available by default. To install these packages, run:

python3 -m pip install regex pysam --user

    Using the “‐‐user” argument will install the packages for the current user only; omit this argument to install those system‐wide (which may require administrative privileges).
  c. Execute the script to identify loci and generate the catalogue.
  This script requires a reference genome as an input parameter (‐‐reference) along with the generated tsv file (‐‐tsv), which can be either “prot3.casecontrol_locus.tsv” or “prot3.outlier_locus.tsv” for case‐control or outlier analysis, respectively. Specify the analysis type (‐‐type) and the maximum *p* value required for significance (‐‐pval). Finally, indicate the destination for the resulting variant catalogue file (‐‐out). By default, STR loci with at least 3 repeats will be searched for and defined. This can be modified with an optional parameter (‐‐repeats).
  i. Option A. Case‐control analysis.


python3 software/tools-for-expansionhunter/findLocus.py --reference reference/hg38.fa --tsv prot3.casecontrol_locus.tsv --type case-control --pval 0.05 --out prot3_casecontrol_variant_catalogue.json

    This command assesses each locus in the “prot3.casecontrol_locus.tsv” file. If the p value is less than the minimum score threshold (0.05), the locus will be included in the creation of the variant catalogue. Loci that do not meet this criterion will be excluded.
  ii. Option B. Outlier analysis.


python3 software/tools-for-expansionhunter/findLocus.py --reference reference/hg38.fa --tsv prot3.outlier_locus.tsv --type outlier --pval 0.05 --out prot3_outlier_variant_catalogue.json

  This command evaluates each locus in the “prot3.outlier_locus.tsv” file and includes it in the variant catalogue creation process if the p value is <.05. Loci that do not meet this criterion will be excluded. In this example, the z score for the top case is 1.62 (one‐tailed p  = .053; not significant). Therefore, no variant catalogue was created, as there were no loci meeting the criteria.
8 Run ExpansionHunter tool to acquire more accurate expansion size.
  a. Install STRs genotyping tool ExpansionHunter. Refer to Basic Protocol 1, step 2, for installation instructions.
  b. Genotype samples using ExpansionHunter.
  Specify the sample BAM file you wish to genotype, such as those with an identified high repeat count. In this example, for outlier analysis the sample is “sampleXXL”, and for case‐control analysis the samples are “sampleXL” and “sampleXXL”.


ExpansionHunter --reads examples/prot3/sampleXXL.bam --reference reference/hg38.fa --variant-catalog prot3_casecontrol_variant_catalogue.json --output-prefix prot3_sampleXXL

  Run the same command again for “sampleXL.bam” for genotyping (change the “‐‐reads” and “‐‐output‐prefix” parameter value to correspond to a different file).
9 View the genotyping results.
  ExpansionHunter generates a VCF and JSON file for each sample. You can examine these files using a text editor or other methods. For instance, the “jq” command‐line tool can be utilized to display the reference coordinates of the locus, coverage, motif, genotype, and genotype confidence interval. 


jq -r '.LocusResults[] | "\(.Variants[].ReferenceRegion) \(.Coverage + 0.5 | floor) \(.Variants[].RepeatUnit) \(.Variants[].Genotype) \(.Variants[].GenotypeConfidenceInterval)"' prot3_sampleXXL.json

  Run the same command again for “prot3_sampleXL.json”.
10 Optional. Annotate the ExpansionHunter output files and determine genetic location of expansions.
  Annotating files will help identify the genetic regions where the STR loci are located and prioritize them. Refer to Basic Protocol 2, step 12, on how to annotate ExpansionHunter's output VCF files.
  In addition to the mentioned annotation instructions, we can also annotate *locus.tsv files using the “‐bed” option with SnpEff. This may be the preferred option; in step 7 of this protocol, we may have excluded some loci because we were unable to identify a repeated region in the reference that contains the repeated motif. Annotating *locus.tsv files will include all loci.
  For example:


java -jar software/snpEff/snpEff.jar closest -ud 0 -bed GRCh38.99 prot3.casecontrol_locus.tsv > prot3.casecontrol_locus_ann.tsv
java -jar software/snpEff/snpEff.jar closest -ud 0 -bed GRCh38.99 prot3.outlier_locus.tsv > prot3.outlier_locus_ann.tsv

### Sample data

The content of “prot3.casecontrol_locus.tsv” (step 6A1): 


contig start end motif pvalue bonf_pvalue counts
chr13 102159593 102164446 AAG 0.0126736593387341 0.0126736593387341 sampleXL:877684.3511304237,sampleXXL:1267596272.2666667




The content of “prot3.casecontrol_motif.tsv” (step 6A2): 


motif pvalue bonf_pvalue counts
AAG 0.0126736593387341 0.0126736593387341 sampleXL:29256.14503768079,sampleXXL:1042509083.7333333




The content of “prot3.outlier_locus.tsv” (step 6B1): 


contig start end motif top_case_zscore high_case_counts counts
chr13 102159593 102164446 AAG 1.62 sampleXXL:1267596272.27 877684.35,1267596272.27




The content of “prot3.outlier_motif.tsv” (step 6B2): 


motif top_case_zscore high_case_counts counts
AAG 1.62 sampleXXL:1042509083.73 29256.15,1042509083.73




The content of both “prot3_casecontrol_variant_catalogue.json” and “prot3_outlier_variant_catalogue.json” (step 7): 


[
{
"LocusId": "chr13-102161574-102161724",
"LocusStructure": "(AAG)*",
"ReferenceRegion": "chr13:102161574-102161724",
"VariantType": "Repeat"
}
]




The content of “prot3_sampleXXL.json” (in step 8): 


{
	"LocusResults": {
	"chr13-102161574-102161724": {
	"AlleleCount": 2,
	"Coverage": 52.45945945945946,
	"FragmentLength": 298,
	"LocusId": "chr13-102161574-102161724",
	"ReadLength": 150,
	"Variants": {
	"chr13-102161574-102161724": {
	"CountsOfFlankingReads": "(1, 2), (2, 3), (3, 1), (7, 2), (8, 2), (9, 1), (10, 2), (12, 3), (13, 1), (14, 4), (15, 2), (16, 4), (17, 1), (18, 2), (19, 1), (20, 1), (21, 3), (25, 2), (26, 1), (27, 2), (28, 2), (29, 1), (30, 2), (31, 1), (32, 1), (33, 2), (35, 2), (36, 2), (37, 2), (38, 2), (39, 1), (40, 2), (41, 1), (42, 1), (44, 1), (45, 1)",
	"CountsOfInrepeatReads": "(50, 28)",
	"CountsOfSpanningReads": "(21, 9)",
	"Genotype": "21/118",
	"GenotypeConfidenceInterval": "21-21/84-130",
	"ReferenceRegion": "chr13:102161574-102161724",
	"RepeatUnit": "AAG",
	"VariantId": "chr13-102161574-102161724",
	"VariantSubtype": "Repeat",
	"VariantType": "Repeat"
	}
	}
	}
	},
	"SampleParameters": {
	"SampleId": "sampleXXL",
	"Sex": "Female"
	}
}




Final results in step 9:

For sampleXXL: 


chr13:102161574-102161724 52 AAG 21/118 21-21/84-130




For sampleXL: 


chr13:102161574-102161724 48 AAG 21/93 21-21/77-119




Columns are as follows: reference coordinates, coverage, motif, genotype and genotype confidence interval.

Final results in step 10 after following instructions in Basic Protocol 2 step 12 and 13 where the annotated VCF file is processed with “extractSnpeffAnn.sh” script: 


Locus: chr13:102161574-102161724 Motif: AAG Genotype: 21/118 CI: 21-21/84-130 Distance: 0 bp Region: Intron (1/4) of transcript ENST00000376131 in gene FGF14



## Support Protocol 2 COMPILING ExpansionHunter Denovo FROM SOURCE CODE AND GENERATING STR PROFILES

This support protocol provides instructions for compiling ExpansionHunter Denovo from source code. Additionally, it provides an example of how to generate an STR profile for individual samples.

### Compiling ExpansionHunter Denovo from source code (Linux or MacOS)

1 Download the source code and compile it in the “software” folder. 


cd software/
git clone https://gitlab.com/andreassh/ExpansionHunterDenovo.git ExpansionHunterDenovo_dir
cd ExpansionHunterDenovo_dir/
 mkdir build && cd build 
cmake -DCMAKE_BUILD_TYPE=Release ../source
make
chmod +x ExpansionHunterDenovo
mv ExpansionHunterDenovo ../../
cd ../..

  On a successful compile, the ExpansionHunterDenovo binary file is located in the “software” folder.

### Generate STR profiles for one sample

2 Generate STR profiles for one sample as follows.


ExpansionHunterDenovo profile --reads examples/prot3/sampleA.bam --reference reference/hg38.fa --output-prefix str-profiles/sampleA

## COMMENTARY

### Background Information

A handful of tools have been developed for genotyping STRs. Some of them can determine alleles longer than the read, such as ExpansionHunter (Dolzhenko et al., 2017) and GangSTR (Mousavi et al., 2019). There are also tools developed to find long expansions and expansions not present in the reference genome, including ExpansionHunter Denovo (Dolzhenko et al., 2020) and STRling (Dashnow et al., 2022). ExpansionHunter, a targeted tandem repeat genotyping tool, has been used for genotyping in several studies, including large‐scale analyses (Chubick et al., 2023; Ibañez et al., 2022). It has demonstrated 97.3% sensitivity and 99.6% specificity compared with a PCR assay (Ibañez et al., 2022) and has also shown greater accuracy than GangSTR and HipSTR (Weisburd et al., 2023). Also, ExpansionHunter includes a companion tool, REViewer (Dolzhenko et al., 2022), which utilizes ExpansionHunter's output data to create read visualization images. These images are crucial for assessing genotyping results and identifying false positives. Additionally, there are other resources and tools designed to assist in analyses using ExpansionHunter, such as the STRipy's resources used in this protocol.

Consequently, our choice of methods allows us to provide a start‐to‐end protocol for various objectives in STR analysis. We demonstrate the use of these tools and other publicly available resources to provide accurate results and an approach to assess the numeric outputs in a visual manner. The Alternate Protocol utilizes the software STRipy (Halman et al., 2022), which is designed for genotyping known pathogenic loci, aligning with the aim of Basic Protocol 1. STRipy incorporates ExpansionHunter as a genotyping tool to ensure accurate results but also offers additional features. These include the capability to genotype long expansions, detect population outliers and reports of diseases linked to the locus and if the repeat size is in a pathogenic range. Additionally, STRipy is packaged as a Docker container, allowing usage in systems where software installation rights are restricted. Thus, the Alternate Protocol provides an easy implementation method to achieve the Basic Protocol 1 objective.

The original version of ExpansionHunter was developed by Illumina and is accessible on their GitHub repository (https://github.com/Illumina/ExpansionHunter). There are a few maintained modifications of the tool available, offering further enhancements and optimizations (https://github.com/bw2/ExpansionHunter) or addressing some known issues with the tool (https://gitlab.com/andreassh/ExpansionHunter). The latter modification is used here, which includes bug fixes and the ability to use it in a genome‐wide mode as it can skip loci that are too close to gaps in the reference genome instead of terminating the whole analysis. In a similar manner, we use a copy of REViewer (https://gitlab.com/andreassh/REViewer) instead of the original (https://github.com/Illumina/REViewer), which has updated libraries to enable successful building of the software from source. Finally, we are using a copy of ExpansionHunter Denovo (https://gitlab.com/andreassh/ExpansionHunterDenovo) instead of the original (https://github.com/Illumina/ExpansionHunterDenovo) as the former addresses an issue with the calculation of outlier *z* scores. For more information about the exact differences, see the repositories.

Apart from the aforementioned software, Basic Protocol 2 uses Tandem Repeats Finder, which is a widely used tool for identifying repeat sequences throughout a genome. With >5000 citations (Tandem repeats finder, see Internet Resources), this tool has demonstrated its utility and offers several parameters that allow users to adjust the tool based on their specific needs, fitting well in this protocol. Finally, Basic Protocol 3 employs ExpansionHunter Denovo to perform a genome‐wide search for repeat expansions, which is a software that has been successfully used in several studies to find disease associated repeat expansions (Pellerin et al., 2023; Rafehi et al., 2023; Scriba et al., 2020; Watanabe et al., 2022).

### Critical Parameters

#### Parameters for ExpansionHunter

##### Sex

By default, ExpansionHunter treats sex as “female”, which considers all chromosomes as diploid. However, when analyzing loci on the X chromosome in male samples, the sex should be specified (sex male). This setting then returns a haplotype instead of a diplotype for such loci.

##### Minimum number of reads required for genotyping

By default, ExpansionHunter requires at least 10 reads to genotype a locus; otherwise, the locus is marked as “LowDepth”. By using the “‐‐min‐locus‐coverage” parameter, this threshold can be lowered, enabling the genotyping of loci with low coverage. Conversely, this threshold can be also increased in order to provide results with higher confidence.

##### Analysis mode

Two analysis modes can be used with ExpansionHunter: “seeking” and “streaming”. By default, “seeking” is used, where the alignment file indexing is used to find (seek) specific reads for analysis. This method is recommended for small catalogues and requires the input BAM or CRAM file to be sorted and indexed.

On the other hand, the “streaming” mode is recommended for large catalogues. In this mode, the input alignment file is read in a single pass, and all variants are analyzed during this operation. This mode does not require the BAM/CRAM file to be indexed. However, while this mode is faster, it also requires significantly more memory (depending on the size of the catalogue) than the “seeking” mode and may result in a different repeat size estimation for repeats longer than the read length.

##### Other

See the documentation at https://gitlab.com/andreassh/ExpansionHunter/‐/blob/master/docs/01_Introduction.md.

#### Parameters for STRipy

##### Type of analysis

STRipy uses a parameter “‐‐analysis”, which can be set to either “standard” (default) or “extended”. In the latter case, for long expansions, STRipy attempts to automatically detect regions in the genome where reads originating from the expansion might be misaligned. This only affects repeats that are longer than the read length, which is usually 150 bp. While the “extended” analysis could bring us closer to determining the true length of the allele, the size can be still underestimated or overestimated (depending on the upstream analysis and whether there are any other expansions with the same motif in the genome that could influence the results).

##### Sex

Similarly to ExpansionHunter, STRipy accepts the parameter “‐‐sex”, which should be used for analyzing loci in X chromosome in male samples.

##### Other

See the documentation at https://gitlab.com/andreassh/stripy‐pipeline.

#### Parameters for tandem repeats finder

Tandem Repeats Finder (TRF) parameters are detailed on their official website (https://tandem.bu.edu/trf/home). In this section, we discuss a few parameters that users might consider adjusting, based on their needs.

The first parameter is “MaxPeriod”. This defines the maximum size of the repeat that the program will identify. For instance, to find only STRs up to 6 bp in length, set the MaxPeriod value to “6”.

A second important parameter is “Minscore”. It sets the minimum alignment score a tandem repeat must achieve to be included in the output. For example, with a default match weight of “2”, setting the Minscore to “50” implies that only tandem repeats with an alignment score of 50 or higher will be considered. This would correspond to a pure tandem repeat sequence of 25 nucleotides without any mismatches or indels (25 nucleotides × match weight of 2  =  50).

To target shorter repeat sequences, the Minscore can be lowered. For instance, a Minscore of “30” with a match weight of “2” targets sequences of at least 15 nucleotides in length (15 nucleotides × match weight of 2  =  30). For example, this finds STR regions that are made of at least five trinucleotide repeats or three pentanucleotide repeats.

Do note that the presence of mismatches affects the score. Using a mismatch penalty of –7, a 15‐nucleotide sequence with a single mismatch would have a score of just 23 (30 – 7). Consequently, it would not be included in the results. To account for potential mismatches or slight motif variations within the tandem repeat, the score has to be adjusted accordingly. For instance, to accommodate a single mismatch within a 15 bp repeat, the Minscore must be decreased by the mismatch penalty, resulting in a new Minscore of 23 (30 – 7). This adjustment would allow for sequences like CAG‐CAG‐CAG‐CAT‐CAG, which has a score of 21.

By default, mismatch and indel penalty score is set to “–7”. This means that longer repeats with more mismatches will be included as well. For example, a pentanucleotide motif AAAAC in AAAAC‐AAAAC‐ACAAC‐ACAAC‐AAAAT sequence results in a score of 30 as well despite there are only two AAAAC motifs present. To avoid those sequences, the penalty score should be higher. For example, if we would use a penalty score of –10 then this sequence score would be 24 and below threshold of 30. Increasing the penalty significantly higher (e.g., to –100), would provide mostly pure STR loci, which is useful when either wanting to discard regions with several mismatches or shorten the length of a longer STR region that has impurities in the start or end. When specifying parameters for TRF, positive values shall be used (i.e., use 100 instead of –100).

In the Basic Protocol 2 the following parameters were used: “2 100 100 80 10 30 6 –h ‐d ‐ngs”, with the following meaning.

Matching weight: 2
Mismatching penalty: 100
Indel penalty: 100
Match probability: 80
Indel probability: 10
Minscore: 30
MaxPeriod: 6
Suppress html output (parameter: ‐h)
Produce a data (.dat) file (parameter: ‐d)
Create more compact .dat file (parameter: ‐ngs)

#### Parameters for ExpansionHunter Denovo

##### Regions

ExpansionHunter Denovo accepts the “‐‐target‐regions” parameter, which allows the use of a BED file to restrict analysis to only the regions specified in the BED file.

##### Other

See the documentation at https://gitlab.com/andreassh/ExpansionHunterDenovo/‐/blob/main/documentation/00_Introduction.md.

#### Parameters for ExpansionHunter Denovo merge

##### Minimum and maximum unit length

“ExpansionHunterDenovo merge” command is used to combine computed STR profiles from each sample in the dataset into a multisample STR profile. This process involves merging individual STR profiles to create a consolidated profile that encompasses all samples.

The two optional parameters (“‐‐min‐unit‐len” and “‐‐max‐unit‐len”) determine the shortest and longest repeat units to consider, respectively. By default, the range is from 2 to 20. However, changing the value of “‐‐min‐unit‐len” from 2 to 3 will exclude all dinucleotide repeats. Similarly, modifying the “‐‐max‐unit‐len”, e.g., to 6, will exclude all motifs longer than that, restricting it strictly to 3 to 6 bp repeat motifs.

##### Parameters for ExpansionHunter Denovo profile

Similar to the “merge” command, minimum and maximum repeat unit lengths can be specified here as well (refer to the previous section for a description). In this context, two additional parameters (“‐‐min‐anchor‐mapq” and “‐‐max‐irr‐mapq”) can be defined, referring to the minimum mapping quality for anchored reads and the maximum for in‐repeat reads, respectively. We recommend using the default value (40) for the minimum MAPQ of an anchor read. However, the maximum MAPQ value for an in‐repeat read could be increased from the default value of 40 to 60, which is the maximum mapping quality of a popular aligner BWA‐MEM. In such cases, in‐repeat reads that align well on some repeated region in the genome will also be included, instead of discarding them. Nonetheless, these values may depend on the specifics of the upstream analysis.

### Understanding Results

We recommend using PCR‐free sequencing data to achieve the best results. This is because PCR can introduce stutter noise (Hauge & Litt, 1993), i.e., reads containing artificial variation in the number of repeats, and potentially results in low coverage regions due to GC‐bias (Aird et al., 2011). For example, loci containing CG‐rich repeats, such as CGG, are particularly prone to GC bias and they may not be genotyped by tools when the coverage is very low or even nonexistent. It is critical to check for error messages when executing any command in these protocols. The presence of such messages may indicate incomplete or unreliable output. All errors must be resolved before moving on to the next steps. See the Troubleshooting section for common problems and solutions.

Completing all steps on the example files in Basic Protocol 1 provides genotyping results for four STR loci. Expansions of ≥50 repeats at the DMPK locus are known to cause myotonic dystrophy type 1 (DM1) (Gutiérrez Gutiérrez et al., 2020). Successful genotyping reveals a heterozygous genotype with one allele having 5 repeats and the other 129. This places the latter within the pathogenic range for DM1, which is an autosomal dominant disease. In the HTT locus, the genotype is 18 and 28 repeats, where the latter is falling in an intermediate range for Huntington's disease (Semaka et al., 2010). The ATXN2 and ATXN10 loci are within the normal range, with homozygous genotypes of 22/22 and 12/12, respectively (Matsuura et al., 2000; Sanpei et al., 1996).

Visual assessment of the reads indicates good coverage at both the DMPK and HTT loci. For HTT, reads spanning the entire STR locus provide an accurate estimate of the repeat length with a tight confidence interval (CI). In the case of the DMPK locus, while the first allele is well‐covered by spanning reads, the second allele lacks such reads because the repeated sequence exceeds the read length. However, several flanking reads are present. One side of those reads comprises the repeat sequence while the other side contains the flanking sequence, which can be used to “anchor” the read to the correct position in the reference genome. The presence of in‐repeat reads (reads consisting solely of the repeat unit) that are visible both in the read alignment image and when inspecting the output VCF or JSON file, indicates that the repeat size exceeds the read length and may even approach or exceed the fragment (paired‐end reads) length. By using both flanking and in‐repeat read information, the estimated allele size was determined to be 129 with a CI of 98 to 154.

Often, expanded repeats will result in a larger confidence interval, as in the example above, because the repeat sequence is much longer than the read length. Determining whether the whole repeat falls within or exceeds the typical fragment length (often ≥300 bp) is challenging with short‐read sequencing data. The presence of numerous in‐repeat reads suggests an expansion longer than the read length, and in‐repeat read pairs (with both reads consisting solely of the repeated sequence) even a longer expansion exceeding the fragment length. However, the challenge lies in the fact that reads consisting completely of repeats align to the best‐matching locations in the genome, which may not be the STR locus that is being analyzed (targeted).

**Figure 8:**
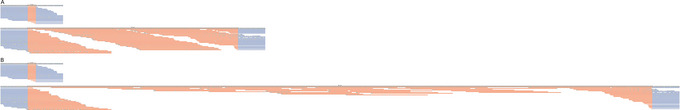
Example read alignments of the same locus genotyped without (**A**) and with (**B)** off‐target regions. By not using off‐target regions (default behavior), the estimated repeat length is limited to the fragment length but having a good coverage across the repeated region (**A**). However, by specifying off‐target regions, it is possible to find and use reads composed of the targeted repeat unit but aligned to an incorrect genomic region, potentially providing a better estimate of the locus length; however, often not well supported by reads (**B**). On the flip side, this approach carries the risk of overestimating the length when using reads not originating from the expansion.

Usually, when genotyping with ExpansionHunter, only paired‐end reads aligning in or near the targeted STR locus are included in the genotyping model, which as a result would exclude in‐repeat read pairs that are misaligned to another location in the genome. For such cases, ExpansionHunter allows specifying “off‐target” regions that can be used for acquiring reads from these areas and use in the genotyping model to aid in estimating the repeat length. For example, with a sequencing depth of 30×, ∼30 misaligned (CAG)_n_ in‐repeat read pairs would indicate a repeat length of >100 repeats (assuming the use of 150 bp paired‐end reads). Generally, we recommend conducting the analysis initially without using off‐target regions. If an expansion is identified that is genotyped to be a few hundred base pairs in length, we recommend the analysis for this particular locus be performed again including off‐target regions. However, one caveat is that if another long endogenous and/or expanded locus with the same motif (e.g., CAG) exists in the genome, then reads originating from those loci that are aligned to the off‐target regions could mistakenly be attributed to the targeted locus. As a result, the use of these wrongly assigned reads can lead to an overestimation of the repeat length. Therefore, off‐target regions should be used cautiously, especially for common motifs like CAG. Figure 8A shows the default method of genotyping of the DMPK locus (good coverage over the locus is visible that provides more confidence that the repeated region is at least of this length). In contrast, Figure 8B illustrates the results when incorporating off‐target regions, which increases the estimated genotype from 129 to 383 repeats, but on the other hand, shows low coverage (therefore, providing less confidence in the determined genotype and in such cases, the confidence interval should be considered).

In Basic Protocol 1, step 4, a variant catalogue was created without off‐target regions, thereby limiting the estimation of long alleles to around fragment length (if any). However, off‐target regions can be easily used when creating the catalogue with STRipy's catalogue creator tool, which contains a list of predefined regions for each locus where reads full of repeats may align. In order to define and genotype a new locus with ExpansionHunter and estimate the length of a long expansion, it is necessary to define off‐target regions. One option involves using previously defined regions for another locus with the same motif. However, this method may not be the best fit as some reads might still be inaccessible. A second approach involves simulating reads from the locus with a long repeat (at least several hundred base pairs to generate in‐repeat read pairs) and then perform alignment to empirically identify the genomic regions where reads misalign, which can then be designated as off‐target regions. Regarding the use of custom loci, we recommend employing the Alternate Protocol, in which the STRipy tool is utilized to automatically identify off‐target regions.

**Figure 9:**
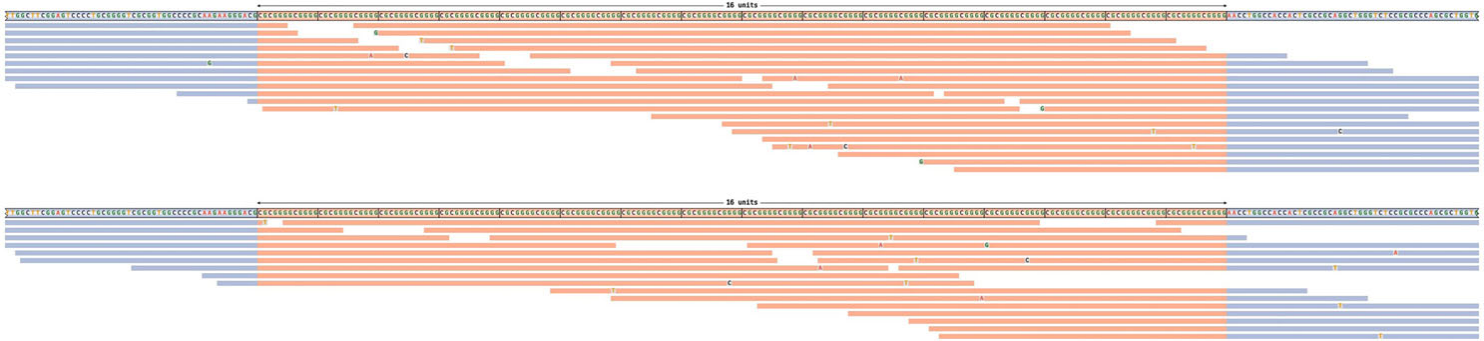
Example of a pathogenic VNTR expansion that is reported as shorter (false negative). Although the pathogenic cutoff for CSTB is 30, a pathogenic sample is estimated to have 16 repeats and is not classified to be in the pathogenic range. This discrepancy is due to limitations in the analysis, which results in an underestimation. Using off‐target regions could provide a better estimate. However, every allele that falls outside the normal population range and exceeds the read length should be flagged for further analysis.

Another challenge in determining whether an allele falls within the pathogenic range occurs when the pathogenic cutoff exceeds the fragment length. For instance, the CSTB locus has a 12 bp VNTR repeat with a pathogenic cutoff of 30 repeats, corresponding to 360 bp (Lalioti et al., 1998). As a result, the estimated genotype using standard analysis (without off‐target reads) is likely to be lower than this threshold, as illustrated in Figure 9. This result does not necessarily mean that the genotype is within the normal range; rather, it suggests that accurate estimation above the pathogenic cutoff is limited by the sequencing technology. In scenarios where the repeated sequence is longer than the read length, it can be suggested to use population frequencies to determine whether the estimated repeat length is an outlier. In this protocol, the output VCF files are annotated with the disease name, inheritance mode, the range into which the repeat sizes fall, and whether the repeat size is an outlier when compared to the population. For this annotation, population data from STRipy's database was used, which is based on genotypes from 2504 individuals in the 1000 Genomes Project cohort and covers various populations. In this annotation process, a population outlier is defined when *z* score ≥3.72 (one‐tailed *p* < .0001). In situations where a locus has a high pathogenic cutoff and the allele is determined to be in the normal or “unknown range” (a category between normal and intermediate/pathogenic, where the literature lacks information), further investigation is highly recommended if the patient exhibits symptoms related to the disease, rather than disregarding the result.

**Figure 10:**
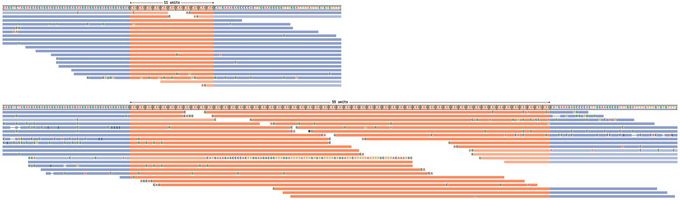
Example read alignments of an STR locus shorter and longer than read length. Reads that span the entire STR region and containing both flanking ends, provide high‐confidence genotyping results (top). When the repeat length exceeds the read length, no spanning reads exist (bottom). Flanking reads, where one end of a read anchors to the flanking region, are used, but which are likely resulting in less accurate genotype estimation.

**Figure 11:**
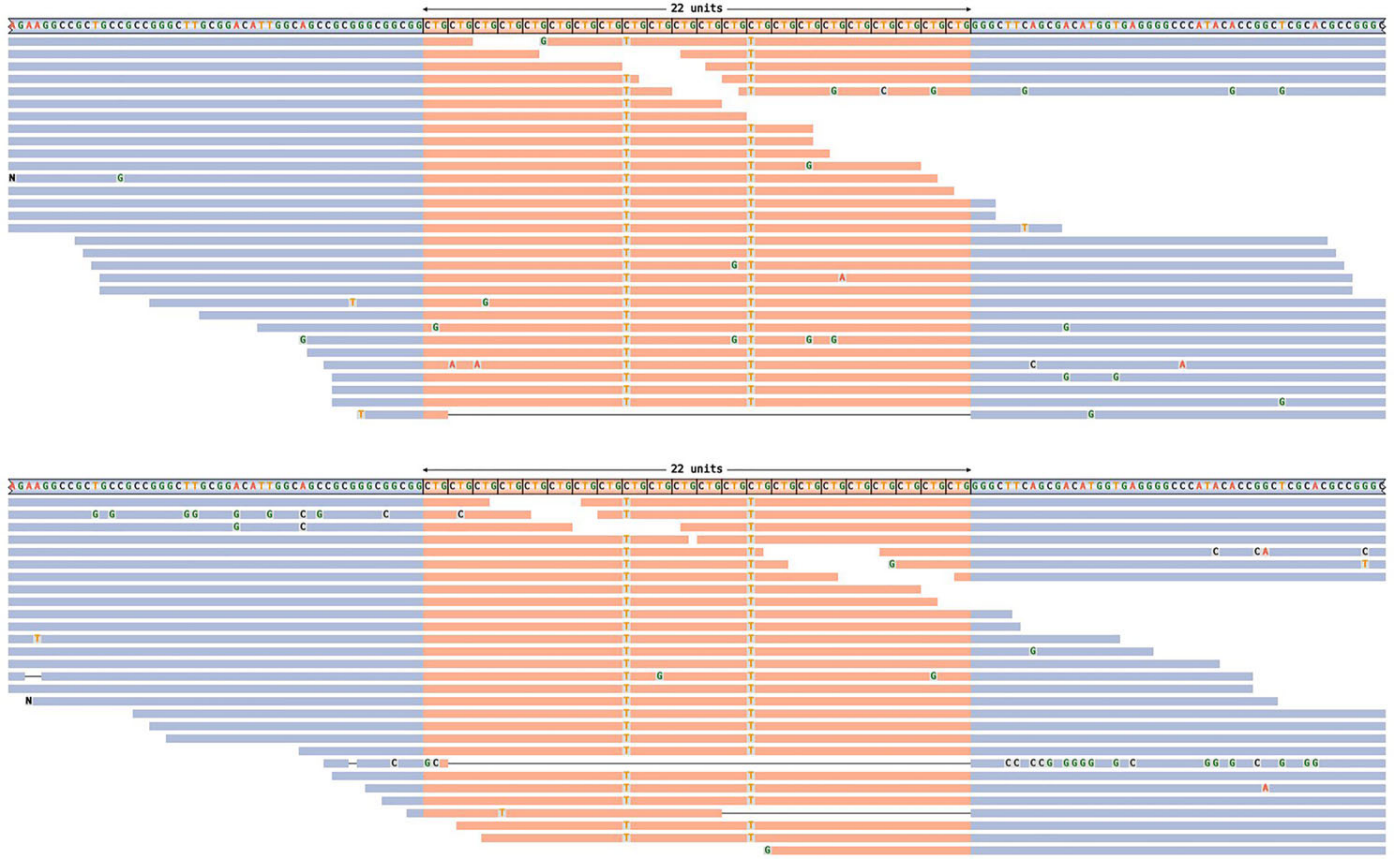
Example read alignments of an STR locus with interruptions. The whole locus length is estimated as 22 units (homozygous); however, there are two instances where CTG is replaced with TTG motif. For some loci, interruptions are known, for others it may not be, which then could have biological importance.

**Figure 12:**
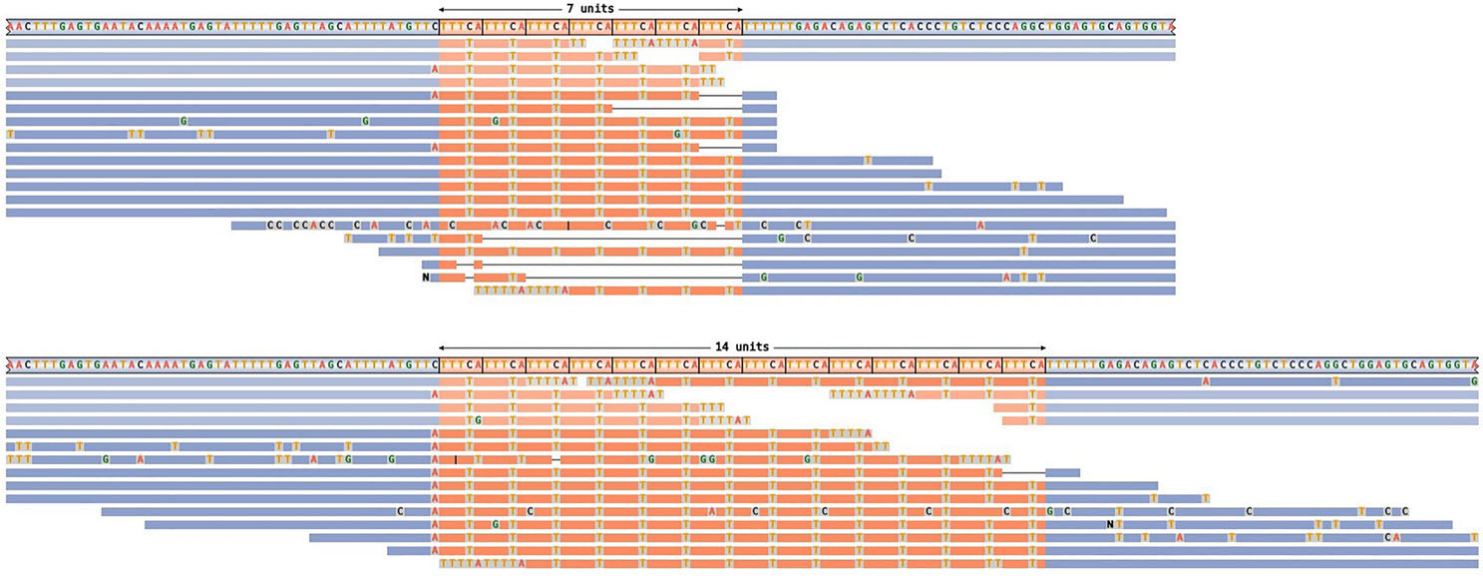
Example of nested type of repeats without the presence of the pathogenic motif. Nested/replaced types of repeats contain a different motif that is not present in the reference genome. When genotyping such loci, even if the pathogenic motif is used, the endogenous repeats will also be genotyped (and reported as mismatches). For these loci, viewing the read alignment is crucial, as it will show whether the pathogenic motif is present or missing (in this case, for the YEATS2 locus TTTTA is endogenous and TTTCA is pathogenic).

As previously illustrated, visual assessment of read alignments aids in assessing the genotyping results. To demonstrate different outcomes, Figure 10 presents a sample where one allele is shorter than the read length, enabling a confident genotype determination using spanning reads, which are also visible for the top allele on the read alignment figure. On the other hand, the second allele exceeds the read length, leading to decreased confidence in length estimation for the reasons discussed earlier. For this allele, no spanning reads are observable. Another point to consider is that a locus may contain sequence variants or interruptions as shown in Figure 11, which could be biologically significant. However, interruptions are a common feature of some STR loci (Bachinski et al., 2009). There is also a type of repeats (so called “replaced/nested”) where affected individuals have repeats that are made of different motifs than what are typically found in healthy individuals and therefore not present in the reference genome. Consequently, when genotyping such “pathogenic” motifs (e.g., targeting TTTCA motif in the YEATS2 locus), the genotyping result likely reflects a genotype for the benign (TTTTA) motif. This occurs because ExpansionHunter counts all motifs with similar size and minor nucleotide differences, resulting in an outcome that indicates the targeted (pathogenic) motifs are present while they are not. Examining the read alignments, such as in Figure 12, can reveal this issue as it becomes evident that the repeated reads are only made of the benign (TTTTA) motif and different from the targeted (TTTCA) motif displayed on top.

**Figure 13:**
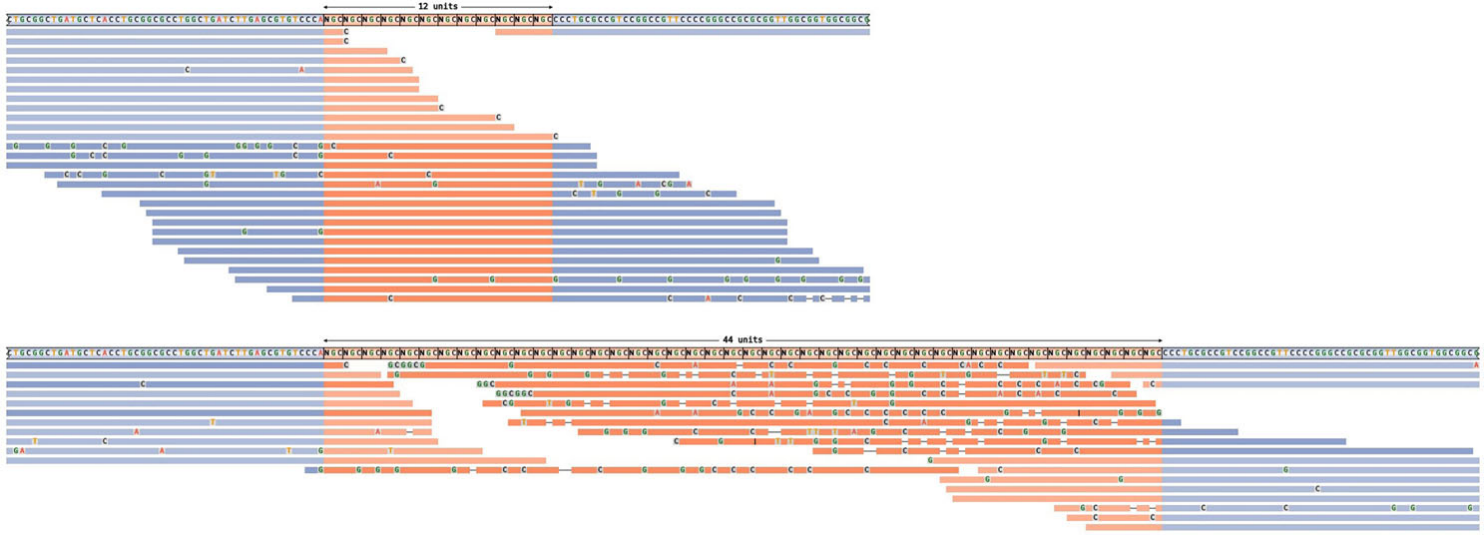
Likely a false positive imperfect GCN sample. The first allele of the ARX_2 locus has high confidence due to several spanning reads, while the second allele has no spanning reads and has only in‐repeat and flanking reads with several nucleotide mismatches and inconsistencies among the reads.

Moreover, read alignment visualizations are recommended as they can also reveal poor alignments. An example is shown in Figure 13, where although the second allele is estimated to fall within the pathogenic range, it is likely a false positive due to inconsistencies in reads and poor alignment. Based on our experiences, this issue can arise frequently with ExpansionHunter when analyzing imperfect GCN repeat loci, which are more challenging to align (e.g., ARX and HOXA13 loci).

In the Alternate Protocol, the STRipy software is used, which also employs ExpansionHunter as its genotyping module. However, further capability to automatically define off‐target regions and genotype long repeats is provided, replacing the process that requires manual specification of off‐targets when using ExpansionHunter independently. Moreover, STRipy uses an updated catalogue of known pathogenic loci, enabling genotyping of any of them in an easy manner with a genotyping report that shows all genotyped loci along with annotations for disease relevance and population outlier status.

In summary, for known pathogenic loci, tandem repeat analysis using short‐read sequencing data serves well as a first‐line screening tool. While it can accurately genotype repeats contained within the read length, it is crucial to know its limitations with longer expansions. In situations where there is an indication of such long expansion exceeding a read or even more, fragment length, it is advisable to visualize the reads and validate the results with a more accurate method.

In Basic Protocol 2, genome‐wide genotyping of STR loci was performed. At first, a total of 1,423,077 STR loci were defined by the TRF tool on the hg38 reference genome. After removing alternate contigs, 1,355,024 loci remain. Filtering out homopolymers leaves 939,963 loci and requiring at least 5 consecutive repeats results in 384,786 loci. This list was used for genotyping the provided example BAM file. Given that the example sample has read coverage for only a small number of loci, this resulted in a very limited number of hits, with all other loci having no coverage. An additional filtering step was applied to remove all loci with no calls or with a low coverage (<10 reads, those loci are marked as “LowDepth” by ExpansionHunter in the VCF file).

Upon examining the genotyping results, one locus (chr19:45770204‐45770264) has a determined genotype of 5/93 CAG repeats (CI 5‐5/93‐146), and the second one (chr4:3074876‐3074933) has a genotype of 18/28 CAG repeats. The first locus corresponds to the DMPK gene and the second to the HTT gene, the same expansions found in Basic Protocol 1 (since the same sample file was used). Upon examining the coordinates of both loci, it becomes apparent that the coordinates determined in this protocol for the DMPK and HTT loci are identical to the manually curated ones used in Basic Protocol 1, demonstrating successful application of the protocol. Regarding genotype, the results for HTT are also the same, while for DMPK, Basic Protocol 2 yields a genotype of 5/93, compared to 5/129 in Basic Protocol 1. The differences arise from the analysis mode used in ExpansionHunter. In Basic Protocol 1, we employed a “seeking” mode for ExpansionHunter that is recommended for smaller catalogues where alignment file indexing is utilized to locate (seek) specific sets of reads for the analysis of each variant. On the other hand, in “streaming” mode, the alignment file is processed in a single pass, allowing for the simultaneous analysis of all variants when the file is being read, which is used for large catalogues to speed up the analysis. As demonstrated here, this may result in differences in genotypes between long alleles using different analysis modes of ExpansionHunter. The reason may lie in the inclusion of extra reads and their mates, which could also potentially impact coverage, fragment size estimates and confidence intervals. When we change the analysis mode from streaming to seeking in Basic Protocol 2, the results for DMPK are identical to Basic Protocol 1 (genotype of 5/129). Therefore, a user might want to consider reanalyzing loci where expansions (over the read length) were discovered by using the “streaming” mode for potentially more accurate genotype results.

As an optional step, the filtered VCF file outputted by ExpansionHunter was annotated using the SnpEff program. Utilizing the “closest” parameter, the tool annotated each locus with the nearest genetic regions to the STR locus. In Figure 6, we can see that for the HTT STR locus (chr4:3074876), the distance to the nearest exon is 0 bp, indicating its location is within an exon. Due to alternative splicing, the STR locus can be part of different transcripts, residing in an exon for one transcript and in an intron for another. Therefore, multiple regions are often included in the annotation, each referring to different transcripts. For DMPK (chr19:45770204), the location is also within an exon. The annotated VCF file also reveals several more loci located within exons and a few in introns.

This protocol is applicable to other organisms as well, provided they have diploid genomes (including plants) and a reference genome is available. The results can be used for characterizing genome‐wide repeats in samples or identifying expansions. However, it is important to note that the determined repeat length is limited by the fragment length. To overcome this limitation and use off‐target regions, the simplest approach is to use STRipy software (see Alternate Protocol) with a custom catalogue option (a BED file with coordinates) that attempts to automatically identify off‐target regions to provide a closer estimate of the repeat length.

In Basic Protocol 3, we employed the ExpansionHunter Denovo (EHdn) computational tool to identify any loci with long expansions in the genome. In contrast to ExpansionHunter or STRipy that use a predefined catalogue, this method conducts a genome‐wide search for tandem repeats (2 to 20 bp motifs by default) where the repeats exceed the read length. It identifies anchored in‐repeat reads, where one read of a pair is anchored at a specific location near the repeat region, while its mate is composed entirely of repeats. Additionally, it searches for in‐repeat read pairs where both reads are made of the repeat unit (e.g., if the repeat unit is CAG, then both reads are composed solely of CAG repeats, albeit with possible interruptions and mismatches). The accompanying scripts for EHdn conduct statistical tests to identify loci and/or motifs significantly increased from the control dataset.

The provided example control dataset included simulated reads at the FGF14 locus, with repeat lengths within the normal range for 15 samples (between 6 and 26 repeats). Additionally, two samples with expansions were simulated: one (sampleXL) with 102 repeats, and the other (sampleXXL) with 500 repeats. The case‐control analysis for this locus, treating both sampleXL and sampleXXL as cases and the other samples without expansions at the locus as controls, successfully identified these two samples as significantly divergent from the control dataset (*p* < .05). However, an outlier analysis using the same dataset only identified sampleXXL as an outlier, but with a lower *z* score (1.62) that was not significant (*p*  = .053) and therefore no variant catalogue was created. Given that statistical significance is influenced by the size of the control dataset, a user might opt to adjust the *p* value threshold upwards (e.g., to 0.1 or even higher) when working with a smaller control dataset to avoid missing true positives.

Since ExpansionHunter Denovo provides only approximate coordinates for the location of the repeated locus, which can be thousands of base pairs long, a helper tool was provided to identify all STR loci within the reported region and create a variant catalogue for the ExpansionHunter genotyping tool. This approach streamlines the process of identifying relevant STR loci and genotyping the significant ones. The genotype estimates for sampleXL were 21/93 (CI 21‐21/77‐119) and for sampleXXL, 21/118 (CI 21‐21/84‐130). While the estimates for the shorter alleles are accurate, the longer allele is underestimated as the simulated length was 500 repeats.

As previously mentioned, the STRipy software can be employed in such cases by using it with a custom catalogue option for genotyping samples with long expansions. For example, based on the analysis conducted on the sampleXXL outside of this protocol, STRipy in “standard” mode provided an identical result for the long allele (118 repeats with a CI of 84 to 130). However, when switching to “extended” mode, a better estimate was obtained: 464 repeats (CI 320 to 498). Also, as discussed previously, using off‐target regions should be applied with caution in real‐life samples.

Figures 8 to 13 demonstrate common examples of read alignments one may encounter.

### Troubleshooting

Some potential errors and their corresponding solutions are outlined in Table 1.

**Table 1:** Troubleshooting the Analysis of Tandem Repeats in Short‐Read Sequencing Data

Problem*^a^*	Possible cause	Solution
Error when running a command or output file is empty/not created	The command is incomplete or contains errors, such as line breaks when copying directly from the PDF file	Review the command to ensure it is correctly formatted as a single line. Check for unintended line breaks or spaces that may have been introduced
Genotyping certain loci does not yield results due to low coverage	PCR amplification used in preparing the library for sequencing can result in GC bias, leaving GC‐rich repeats with little or no coverage	Use PCR‐free sequencing to genotype all loci with confidence
		Use the “‐‐min‐locus‐coverage” parameter with ExpansionHunter to specify a value lower than the default of 10 (e.g., to 3) to enable genotyping of loci with very low coverage (note that these results must be carefully examined along with read visualizations)
ExpansionHunter returns an error: “*Flanks can contain at most 5 characters N but found X Ns*”	The locus currently in analysis is too close to the chromosome ends and contains N characters; this can happen with the original version of ExpansionHunter	Use an updated version of ExpansionHunter from https://gitlab.com/andreassh/ExpansionHunter repository that skips such loci by default
ExpansionHunter returns an error: “*X is not a path to an existing file*”	Wrong input file (BAM, FASTA or JSON) for ExpansionHunter is used	Check the location of input files and ensure that the correct path is specified for the tool and correct parameters used
Running ExpansionHunter on the whole genome results in an “oom_kill event” or a similar issue	Not enough RAM was allocated	Allocate more memory, usually >64 GB, preferably >100 GB
ExpansionHunter returns an error: “*Error loading locus X: Invalid contig name X”*	Sample (BAM or CRAM) file does not include the locus (usually chromosome) that was specified in the variant catalogue file; this can also happen when chromosome names in variant catalogue contain “chr” but not in the aligned file (or *vice versa*)	Check the variant catalogue file and remove the locus manually or use a different (correct) reference genome to prepare files
Running “SnpEff closest” is stuck	The database does not exist	Make sure you have downloaded the database (e.g., GRCh38.99) and are using the same version when running SnpEff

^*a*^ “X” denotes a text that can vary.

### Author Contributions

**Andreas Halman**: Conceptualization; investigation; methodology; project administration; resources; software; visualization; writing—original draft; writing—review and editing. **Andrew Lonsdale**: Software; writing—review and editing. **Alicia Oshlack**: Methodology; resources; supervision; writing—review and editing.

### Conflict of Interest

The authors declare no conflict of interest.

## Data Availability

The data that support the protocol are openly available on Zenodo at http://doi.org/10.5281/zenodo.10872070.
